# Non-Invasive Extracellular Vesicle Biomarkers in Endometriosis, Molecular Signatures Linking Pelvic Inflammation, Oocyte Quality, and IVF Outcomes

**DOI:** 10.3390/cimb47110956

**Published:** 2025-11-17

**Authors:** Charalampos Voros, Fotios Chatzinikolaou, Ioakeim Sapantzoglou, Georgios Papadimas, Spyridon Polykalas, Despoina Mavrogianni, Aristotelis-Marios Koulakmanidis, Diamantis Athanasiou, Vasiliki Kanaka, Maria Kanaka, Kyriakos Bananis, Antonia Athanasiou, Aikaterini Athanasiou, Ioannis Papapanagiotou, Dimitrios Vaitsis, Charalampos Tsimpoukelis, Maria Anastasia Daskalaki, Marianna Theodora, Nikolaos Thomakos, Panagiotis Antsaklis, Dimitrios Loutradis, Georgios Daskalakis

**Affiliations:** 1 Department of Obstetrics and Gynecology, ‘Alexandra’ General Hospital, National and Kapodistrian University of Athens, 80 Vasilissis Sofias Avenue, 11528 Athens, Greece; kimsap1990@hotmail.com (I.S.); depy.mavrogianni@yahoo.com (D.M.); aristoteliskoulak@gmail.com (A.-M.K.); vasilikikanaka@outlook.com (V.K.); maira.kanaka24@gmail.com (M.K.); tsimpoukelischa@gmail.com (C.T.); md181341@students.euc.ac.cy (M.A.D.); martheodr@gmail.com (M.T.); thomakir@hotmail.com (N.T.); panosant@gmail.com (P.A.); gdaskalakis@yahoo.com (G.D.); 2 Laboratory of Forensic Medicine and Toxicology, School of Medicine, Aristotle University of Thessaloniki, 54124 Athens, Greece; fotischatzin@auth.gr (F.C.); loutradi@otenet.gr (D.L.); 3 Athens Medical School, National and Kapodistrian University of Athens, 15772 Athens, Greece; dr.georgepapadimas@gmail.com (G.P.); spyridonpolykalas@hotmail.com (S.P.); gpapamd@hotmail.com (I.P.); vaitsisdim@gmail.com (D.V.); 4 IVF Athens Reproduction Center, 15123 Maroussi, Greece; diamathan16@gmail.com (D.A.); antoathan16@gmail.com (A.A.); diamathan17@gmail.com (A.A.); 5 King’s College Hospitals NHS Foundation Trust, London SE5 9RS, UK; kyriakos.bananis@nhs.net; 6 Fertility Institute-Assisted Reproduction Unit, Paster 15, 11528 Athens, Greece

**Keywords:** endometriosis, extracellular vesicles, exosomes, microRNA, uterine fluid, follicular fluid, menstrual blood, endometrial receptivity, biomarkers, in vitro fertilization, assisted reproductive technology

## Abstract

Endometriosis impairs fertility by interfering with ovarian function, embryonic development, and endometrial receptivity. Extracellular vesicles (EVs) are recognised as non-invasive biomarkers that may indicate biological processes based on their lipid, protein, and microRNA composition. This narrative review synthesises current data on extracellular vesicle (EV) signatures in serum/plasma, menstrual blood, follicular fluid, and uterine fluid in endometriosis patients using assisted reproductive technology (ART). We highlight critical EV-mediated processes, such as progesterone signalling, fibrosis, angiogenesis, inflammation, and metabolism, and their associations with oocyte competence, embryo development, and implantation. Certain EV-miRNA profiles, including miR-22-3p, miR-320a, the miR-200 family, and miR-145-5p, have shown use for diagnostic and prognostic purposes in various investigations. These characteristics are associated with live birth, implantation, and blastocyst quality. We propose a clinical framework that incorporates (i) menstrual-blood EVs for non-invasive endotyping, (ii) serum/plasma EV profiling for baseline risk stratification, and (iii) pre-transfer uterine-fluid EV evaluation to inform embryo-transfer decisions. Translation requires standardisation, cycle phase control, and prior validation. EVs may serve as a beneficial instrument for personalised in vitro fertilisation operations for ladies experiencing infertility due to endometriosis.

## 1. Introduction

Endometriosis is a chronic, estrogen-dependent inflammatory condition marked by the presence of ectopic endometrial-like tissue, which affects pelvic immune metabolic balance and impairs fertility via interconnected pathways involving the ovarian, embryonic, and endometrial systems [1]. Oxidative stress, excessive cytokines, and disrupted angiogenesis impair granulosa cumulus signalling in the ovaries, undermining mitochondrial function and meiotic integrity, which therefore reduces oocyte competence [2]. Aberrant paracrine signals and an inflammatory milieu may inhibit blastocyst development and cleavage dynamics at the embryonic stage. Progesterone resistance, extracellular matrix (ECM) remodelling, and impaired immune surveillance in the endometrium obstruct communication between the embryo and the endometrium, thereby compromising the implantation window [3]. Non-invasive biomarkers that reflect this biology and support informed decision-making for assisted reproductive technology (ART) are still insufficiently established, despite progress in imaging and laparoscopy that improve detection and staging [4].

Extracellular vesicles (EVs), including apoptotic bodies, microvesicles (about 100–1000 nm), and tiny EVs/exosomes (approximately 30–150 nm), have emerged as significant communicators and biomarkers of disease conditions [5]. Their lipid bilayer prevents enzymes from degrading a substantial array of microRNAs (miRNAs), other non-coding RNAs, mRNAs, proteins, lipids, and metabolites. This facilitates their identification in fluids proximal to the clinic, such as uterine or uterine-luminal fluid, and in accessible matrices, including serum, plasma, and menstrual blood [6]. The vesicular “barcode” is significant since it indicates both lesion activity and normal endometrial function. EV cargo is particular to the originating cell and is continuously altered by inflammation, steroidal signalling, hypoxia, and metabolic stress factors characteristic of endometriosis. Besides passive reporting, EVs actively modify the cells to which they are directed. Via vesicular miRNAs and proteins, they may alter trophoblast adhesion and invasion, induce stromal cell decidualisation, promote endothelial cell sprouting, and modulate macrophage polarisation. Due to their dual role as both effectors and reporters, EVs may serve as a single platform for diagnosing, staging, and forecasting infertility associated with endometriosis [7].

EVs have three specific benefits for ART decision-making. Initially, biological specificity: EV cargo delineates lesion/eutopic signals and immune-stromal interactions that directly affect oocyte quality, embryo viability, and receptivity, as opposed to soluble indicators [8]. Secondly, serial non-invasive sampling involves the collection of uterine fluid samples at the moment of embryo transfer, providing a temporally aligned representation of the implantation window, while peripheral and menstrual blood may be obtained many times during the cycle [9]. Third, pathway-level interpretability: recurring EV-derived signals converge on controllable pathways, such as NF-κB/JAK-STAT (inflammation), TGF-β/SMAD and PI3K/AKT (fibrosis, motility, metabolism), and NOTCH and WNT (embryonic and endometrial patterning) [10]. This creates the opportunity to associate biomarkers with specific adjuvants or timing methods, including receptivity-guided transfer, adjuvant anti-inflammatory or antioxidant protocols, or personalised stimulating approaches. EVs are well positioned to bridge clinical triage and discovery biology. This entails determining the ideal period for endometrial receptivity, evaluating candidates for rapid IVF vs. pre-treatment optimisation, and customising stimulation and transfer methods to improve implantation and live birth rates [11].

### 1.1. Molecular Pathology of Endometriosis Through the EV Lens

This section presents a mechanistic framework that synthesises current information to clarify the connection between EV-mediated signalling and the biology of endometriosis and reproductive failure, rather than presenting unique experimental data. Analysing EVs reveals that the molecular pathology of endometriosis comprises a highly interconnected, self-reinforcing signalling network that spans from the lesion niche to systemic circulation, thereby offering feedback that exacerbates disease characteristics [12]. Endometriotic implants serve as active secretory centres in the peritoneal cavity, where inflammatory signals, hypoxia, and iron-mediated oxidative stress from cyclic bleeding co-activate HIF-1α, NF-κB, and AP-1. This enhances EV generation via ceramide/nSMase2 pathways, Rab27a/b–ARF6 trafficking mechanisms, and ESCRT complexes (TSG101/ALIX) [13]. hnRNPs and YBX1-dependent sorting machinery preferentially incorporate the complex “barcodes” of microRNAs, long non-coding and circular RNAs, mRNAs, proteins, lipids, and oxidised metabolites into the exported tiny extracellular vesicles. Recipient cells, such as fibroblasts, endothelial cells, dendritic cells, mesothelial cells, peritoneal macrophages, or caveolae, internalise these vesicles by clathrin-mediated endocytosis, macropinocytosis, tetraspanin integrin contacts, or heparan-sulphate proteoglycan docking [14]. Vesicular miRNAs targeting the STAT3/SOCS1, PTEN, and granzyme pathways augment CCL2/CCR2-mediated monocyte influx, diminish NK and CD8^+^ cytotoxicity, and facilitate a transition in macrophages towards an M2-like, pro-angiogenic phenotype [15]. The system is irrevocably activated and cannot be reverted to its normal state by the new cytokine milieu (IL-6, IL-8, TNF, and GM-CSF) in conjunction with complement fragments and procoagulant microparticles. This maintains elevated inflammatory levels, which aid in tissue remodelling, pain perception, and immune evasion [16].

EV-mediated communication establishes the neurovascular framework that prolongs pain and lesions. It also facilitates the growth of new blood vessels and nerve cells. Vesicular MMPs or MMP-inducing signals modify the basement membrane and interstitial matrix to facilitate vessel ingrowth. Extracellular vesicle-derived miRNA clusters (e.g., miR-21/miR-132) and proteins (VEGFA co-regulators, angiopoietins) stimulate the PI3K/AKT–eNOS and MAPK/ERK signalling pathways in endothelial cells, enhancing migration, proliferation, and lumen development [17]. The concurrent transmission of neurotrophic factors (NGF/BDNF) and guidance-pathway modulators (semaphorin/neuropilin, netrin, Slit/Robo) enlists and stabilises aberrant sensory and sympathetic filaments [18]. Vesicular signals enhance the sensitisation of nociceptors via TRPV1 and Nav1.8, and facilitate neuro-immune connections that perpetuate hyperalgesia. Platelet-derived extracellular vesicles containing tissue factor and thromboxane exacerbate microthrombi and ischemia–reperfusion cycles, hence intensifying hypoxia and the release of extracellular vesicles [19]. Conversely, stromal and mesothelial cells exposed to lesion-derived extracellular vesicles enhance COX-2/PGE_2_ and endothelin signalling, hence intensifying vasodilation and pain signalling. The neurovascular–inflammatory loop establishes a molecular foundation for the correlation among lesion vascular density, nerve fibre density, and pain intensity, directly associating vesicle cargo with clinical manifestations like dyspareunia and dysmenorrhea [20].

EV-driven systems that integrate transcriptional reprogramming and mechanotransduction regulate fibrotic remodelling, adhesion creation, and invasive growth. Endometrial stromal cells and peritoneal fibroblasts elevate the levels of collagen I/III, fibronectin, periostin, and LOX via TGF-β/SMAD2/3 and YAP/TAZ–TEAD activation [21]. This increases the stiffness of the extracellular matrix and fortifies crosslinks that are resistant to degradation. EV miRNAs that decrease PTEN and RhoGDI augment RhoA/ROCK activity to promote the development of actin stress fibres and focal adhesions via FAK/Src. This, thus, promotes states akin to epithelial–mesenchymal transition and mesothelial-to-mesenchymal transition, facilitating cellular passage over the mesothelial barrier [22]. Vesicular integrins may enhance organotropism and selective adhesion in the peritoneal milieu; nevertheless, imbalanced MMP-2/MMP-9 and TIMP-1/TIMP-2 ratios whether conveyed directly in vesicles or triggered by vesicular signals diminish matrix thresholds for invasion [23]. These structural alterations not only entrap pelvic organs and induce adhesions, but they also modify the stiffness of the surrounding region. This stimulates nuclear mechanosensors (LINC complex, lamin A/C) and epigenetic modifiers, which stabilise profibrotic transcriptional states and transform acute inflammatory damage into chronic architectural illness [24].

EV cargo modulates cellular energy utilisation and mitigates oxidative stress at the metabolic and redox interface, ensuring that cellular metabolism aligns with the demands of migration, growth, and survival under adverse conditions [25]. Vesicular miRNAs that inhibit NRF2, SOD2, PRDXs, or mitochondrial quality-control genes regulate reactive oxygen species levels. This promotes reactions to DNA damage, which paradoxically facilitates invasion and evasion of the immune system [26]. Menstrual-blood EVs prominently include miR-4443, which links PI3K/AKT signalling to acetate absorption by ACSS2, therefore replenishing acetyl-CoA reserves for SREBP1-mediated lipogenesis and histone acetylation (e.g., H3K27ac) [27]. The relationship between metabolic flux and chromatin accessibility, together with the expression of genes facilitating cellular movement, is established. In addition to miRNAs, extracellular vesicles may also transport sphingolipids, oxidised phospholipids, and functional mitochondrial components [28]. These entities may enhance oxidative phosphorylation in recipient cells or, following injury, activate TLR9 and cGAS-STING to intensify sterile inflammation. Simultaneously, alterations in glycolysis and glutaminolysis induced by EV signalling provide biosynthetic precursors and facilitate redox buffering via NADPH [29]. The modulation of adipocyte and hepatocyte metabolism by extracellular vesicles may contribute to the systemic metabolic profile seen in certain patient subgroups. To promote lesion persistence, various levels of metabolic reconfiguration interact with angiogenesis and fibrosis to maintain cellular energetics, matrix integrity, and nutrition transport in unison.

In endometriosis, progesterone resistance and epigenetic drift are emergent features arising from extended extracellular vesicle-mediated contact between eutopic and ectopic tissues, rather than being separate qualities [30]. Vesicular cargos that alter steroid receptor chaperones and co-regulators (FKBP52, NCOA/NCOR families) and chromatin effectors (DNMT1/3A, TETs, EZH2, and HDACs) modify enhancer-promoter landscapes, disrupt the equilibrium of PR-A/PR-B, and attenuate decidualisation genes such as IGFBP1 and PRL. The increase in miR-145-5p in the uterine fluid of women with endometriosis significantly inhibits NOTCH components in human and mouse trophectoderm, consequently delaying or preventing blastocyst development and reducing implantation competence [31]. EVs derived from the endometrium and lesions include miRNAs and morphogens that facilitate sticky and receptive conditions inside the uterine lumen. Mid-secretory signatures (e.g., miR-30d, miR-200 family) are associated with receptivity pathways including LIF, ITGβ3, and osteopontin [32]. Multi-omics analyses indicate that UF-EV miRNomes and transcriptomes monitor alterations in the endometrial phase. These discoveries provide a framework for pre-transfer triage that incorporates both embryo-intrinsic and endometrium-intrinsic readiness, allowing a temporally synchronised, minimally intrusive molecular evaluation of the implantation window. These findings can be consistently replicated over time without requiring additional processes.

Extrapelvic EVs influence the whole body, perhaps explaining symptoms not localised to the pelvis and underscoring the use of peripheral biomarkers in forecasting ART outcomes. Platelet microvesicles convey membranes rich in tissue factor and phosphatidylserine, which enhance thrombin synthesis, whereas neutrophil-targeting vesicles may promote NETosis, intensify inflammatory microthrombi, and modify nociceptive pathways [33]. Circulating extracellular vesicles from lesions, eutopic endometrium, platelets, and leukocytes engage with vascular endothelium to stimulate Weibel Palade body release, enhance VCAM-1/ICAM-1 expression, and augment permeability. In susceptible individuals, interactions between sensory neurones and glial cells possibly involving the passage of extracellular vesicles across fenestrated endothelium may activate central sensitisation pathways [34]. From a reproductive perspective, this circulating extracellular vesicle environment provides easily obtainable, progressively quantifiable indicators: Menstrual blood and UF-EV signatures provide lesion-proximal and endometrium-proximal information, respectively, while serum/plasma EV miRNA arrays have shown considerable effectiveness in predicting pregnancy and delivery in ART cohorts with endometriosis [35]. The findings collectively endorse a unified model in which EVs amalgamate inflammation, fibrosis, angiogenesis, neurogenesis, metabolism, and epigenetic regulation into a singular, dynamically evolving communication system that converts localised pathology into systemic effects, ultimately influencing endometrial receptivity, oocyte competence, and embryo viability, while also offering significant targets for diagnosis and treatment in personalised IVF care [36].

### 1.2. Reproductive Axes Impacted by EVs: From Follicle to Implantation

An EV-focused viewpoint clarifies the effects of endometriosis on the ovarian microenvironment, in conjunction with the established repercussions of peritoneal inflammation. Vesicles secreted by granulosa, cumulus, theca, endothelial, and immune cells in FF regulate redox balance, mitochondrial biogenesis, steroidogenesis, and gap-junctional signalling within the COC [37]. Oxidative stress resulting from iron and cytokine overflow in endometriosis, particularly in the presence of endometriomas, alters the composition of EVs to incorporate miRNAs and proteins that inhibit NRF2/SOD2 defences, impede mitochondrial renewal mediated by PGC-1α, and disrupt the KITL–KIT and GDF9–BMP15 pathways, which are crucial for cumulus expansion [38]. The ingestion of these vesicles by granulosa cells, via clathrin/caveolae or heparan-sulphate docking, undermines meiotic spindle integrity and chromosomal segregation by diminishing ATP availability and enhancing mitochondrial depolarisation. Extracellular vesicle-derived modulators of VEGF/ANGPT signalling exacerbate hypoxia and promote glycolysis in the microenvironment, while also altering perifollicular angiogenesis by restricting the supply of oxygen and nutrients [39]. These alterations result in decreased MII yield, impaired oolemma competency, and inadequacies in future embryo quality. This biology indicates two translational activities concerning ART: (i) Establishing baseline serum/follicular fluid extracellular vesicle panels to identify high-oxidative, low-mitochondrial-reserve follicles that may benefit from antioxidant adjuvants or tailored stimulation; and (ii) Monitoring extracellular vesicle signatures during stimulation to determine the optimal timing for trigger initiation or to contemplate a freeze-all strategy when the cumulus-oocyte complex “transcript” appears unfavourable [40]. Hormonal exposure and illness progression might complicate the interpretation of EV analyses. Every prediction model must include cycle-phase and medication information.

UF-EVs serve as proximate chemical signals that influence initial developmental decisions. Their payload infiltrates the zona-enclosed embryo via endocytosis and membrane fusion. This introduces morphogens and miRNAs that enhance metabolic programming, inner cell mass distribution, and trophectoderm polarity [41]. An illustrative instance of the UF-EV composition biassing towards anti-implantation signals in endometriosis is miR-145-5p, which is increased in the uterine lumen of afflicted women and suppresses NOTCH pathway components in both murine and human embryos [31]. This impedes compaction, compromises trophoblast integrity, and diminishes the rates of blastocyst formation. This represents an embryo-intrinsic bottleneck that remains consistent even in oocytes that seem “healthy.” Besides NOTCH, vesicular regulators of WNT, Hippo (YAP/TAZ), and integrin signalling can modify glucose-lipid partitioning and misdirect adhesion machinery (e.g., ITGβ3, trophinin), resulting in suboptimal morphokinetics evident in time-lapse imaging (e.g., delayed tSB, diminished blastocyst expansion grades). These results redefine the “unexplained” variability in IVF outcomes linked to endometriosis: embryonic potential is affected by the embryo’s interactions with EVs, rather than being exclusively determined by the oocyte. Practical implications include evaluating time-lapse anomalies via uterine signals, prioritising cryopreservation and improving the endometrial environment instead of promoting fresh transfers when UF-EVs suggest an anti-implantation environment, and acquiring UF samples immediately prior to transfer to characterise embryo-facing EV cargo. During certain cycles, choices guided by embryonic viability may affect techniques for transferring blastocysts vs. cleavage-stage embryos, since susceptibility may differ between day-3 and day-5 stages [42].

The WOI is governed by coordinated epithelial, stromal, vascular, and immunological mechanisms that determine receptivity. Multi-omics analyses indicate that UF-EVs elucidate the alterations in endometrial tissue during the menstrual cycle [32]. Mid-secretory vesicles are abundant in immunological and adhesion surface markers, including as CD56, CD45, CD3, and CD142, as well as miRNA panels, particularly the miR-200 family and miR-30d. These markers and panels together regulate trophoblast communication, stromal decidualisation, and epithelial adhesion. Endometriosis disturbs this harmony [43]. Vesicular miRNAs diminish adhesion and apposition via modulating the LIF–STAT3 pathway, inhibiting decidual genes (such as IGFBP1/PRL), and altering the glycocalyx composition of the epithelium. The peri-implantation niche is progressively skewed towards inflammatory and microthrombotic conditions that obstruct the infiltration of EV-encoded tissue factor and coagulation signals. In clinical environments, UF-EV-based models have been used to differentiate between implantative and non-implantative endometrium. These models could be employed immediately before embryo transfer to aid women with endometriosis in making decisions about proceeding, selecting adjuvants (such as anti-inflammatory medications, anticoagulant microdosing, or decidualisation modulators), and adjusting luteal progesterone exposure to avert a misaligned window of implantation. Effective management or modelling of pre-analytics (sample techniques, dilution, blood contamination), hormonal environment (combined oral contraceptives, progestins, GnRH analogues), and lesion phenotype/stage is essential, since these factors substantially alter UF-EV cargo [44]. To coordinate embryo insertion with a molecularly conducive endometrium, an efficient algorithm would include the following elements: (1) Baseline serum extracellular vesicle screening for global inflammatory and angiogenic burden (2) cycle-phase calibrated ultrafiltration extracellular vesicle profiling during the window of implantation to ascertain receptivity (3) decision criteria that initiate delayed transfer upon detection of anti-implantation signals (e.g., elevated miR-145-5p, diminished miR-30d/miR-200b).

### 1.3. Why EVs Are Ideal Non-Invasive Biomarkers in Endometriosis-Associated Infertility

Extracellular vesicles get molecular signals from certain tissues associated with endometriosis-related infertility, including the eutopic endometrium, ectopic lesions, and immunological and vascular cells inside the pelvic niche. Sequence motifs and RNA protein chaperones actively sort extracellular vesicle cargo rather than just partitioning it, resulting in a miRNA/protein “barcode” that records the cell’s origin and activation status. This results in enhanced disease specificity relative to several soluble indicators that reflect downstream inflammation without clarifying processes or integrating information from diverse sources [45]. Serum EV panels may differentiate between inflammatory-fibrotic signals associated with endometriosis and those related to PCOS or pelvic infection. Menstrual-blood extracellular vesicles provide insights into lesions and the endometrium non-invasively [37]. UF-derived EVs, collected during the implantation window, indicate receptivity programmes that conventional serum indicators cannot identify. The biology that renders EVs valuable for comprehension also facilitates their use by connecting panels to pathways (such as NOTCH, TGF-β/SMAD, and PI3K/AKT) and associating them with certain adjuvants or timing techniques in ART [46].

EV membranes protect proteins and nucleic acids against RNases and proteases that might degrade free circulating analytes, particularly in small-volume uterine specimens. This physicochemical protection enables the reliable detection of microlitres of uterine fluid, minimally processed menstrual blood eluates, or standard serum samples [47]. It maintains analytical performance while facilitating long-term sampling during a stimulation cycle. To ensure reproducibility and comparability across cohorts, rigorous normalisation (exogenous spike-ins combined with endogenous reference EV-miRNAs), orthogonal characterisation (NTA/EM/Western blot or bead-based flow cytometry), and MISEV-compliant pre-analytics (expedited processing, temperature regulation, and platelet removal) are employed. These attributes render EVs an optimal selection for clinical laboratories using tailored NGS panels or qPCR, where lot-to-lot consistency and analytical sensitivity are paramount [48].

Numerous complementary, patient-centric resources for EVs exist to address various clinical enquiries. Obtaining serum or plasma samples for baseline risk assessment and therapy monitoring is straightforward, cost-effective, and feasible to do serially [49]. Menstrual blood offers a distinctive, non-invasive perspective on endometrial and lesion biology, enabling screening or monitoring without the need for clinic appointments. Uterine or uterine luminal fluid, obtained in the clinic immediately before to embryo transfer, provides a synchronised evaluation of receptivity and embryo-facing signals, facilitating prompt go/no-go determinations. When available, peritoneal fluid offers a mechanistic basis for communication between the immune system and lesions [50]. By integrating various matrices, physicians may assess the biological stage of the illness enhance diagnosis or endotyping, and verify implantation readiness within the same patient. This makes the operation rational, less invasive, and congruent with conventional IVF protocols.

EVs are not only essential in detecting endometriosis, but they also monitor critical endpoints relevant to IVF. Serum sEV-miRNA signatures demonstrate strong discriminative effectiveness (AUCs > 0.8) in predicting clinical pregnancy and delivery in impacted ART populations, whereas UF-EV concentration indicates mid-secretory endometrial conditions that regulate implantation [51]. The profiles of UF-EV miR-200 and miR-30d are associated with adhesion and decidual pathways that regulate receptivity. Vesicular regulators like as miR-145-5p elucidate the suboptimal functionality of a blastocyst by inhibiting NOTCH signalling. This convergence enables EV panels to diagnose diseases (including their existence and phenotype), choose treatments, and formulate prognoses [52]. For example, additional transfers may be sanctioned when UF-EV signatures indicate a favourable period, freeze-all may be delayed when anti-implantation signals are robust, or adjuvants may be modified if serum EVs exhibit a significant inflammatory or fibrotic burden. With appropriate calibration, decision thresholds, and validation against definitive outcomes (MII yield, usable blastocyst rate, implantation, clinical pregnancy, and live birth), EV assays can assist individuals with endometriosis-related infertility in making informed decisions regarding the timing of conception, selection of embryos, and ultimately, the implantation of embryos [53].

This study seeks to address current gaps by synthesising methodological, clinical, and biological findings into a unified translational paradigm. We (i) elucidate the molecular mechanism by which EVs affect endometriosis pathology along the ovarian, embryonic, and endometrial axes that collectively influence fertility potential (ii) assess the diagnostic and prognostic effectiveness of EV-based biomarkers in easily accessible non-invasive matrices, emphasising the impact of biological covariates such as hormonal environment and pre-analytical factors and (iii) propose a clinically applicable framework for the utilisation of EVs in personalised IVF treatment. The focus is on immediate prospects, including serum-EV miRNA signatures for predicting pregnancy and UF-EV profiling for assessing endometrial receptivity, while detailing the necessary validation protocols, multicentre harmonisation efforts, and computational modelling for their integration into future ART workflows. This narrative review aims to redefine EVs as a transformative therapeutic interface between reproductive medicine and molecular diagnostics, rather than just a mechanistic curiosity. The next section provides a conceptual synthesis of the key molecular processes via which EV signalling influences endometriosis disease and fertility potential, therefore connecting cellular-level EV biology with clinical reproductive outcomes.

## 2. Search Strategy and Literature Selection

This is a concise overview of the search methodology and inclusion criteria used to identify papers concerning extracellular vesicles and reproductive outcomes associated with endometriosis. This ensures that the literature coverage is explicit and reproducible.

This narrative review focusses on the ramifications of ART results, covering recent research on EVs in infertility associated with endometriosis. To improve rigour and repeatability, we used a predefined, transparent methodology, notwithstanding its initial absence of registration as a systematic review. We searched for items published from 1 January 2018 to 30 September 2025, in PubMed, Scopus, and Web of Science. When appropriate, we used prior fundamental research to contextualise processes or methodologies (for instance, EV biogenesis or MISEV guidelines). The search phrases included “extracellular vesicles,” “small extracellular vesicles,” “exosomes,” “microvesicles,” “endometriosis,” “infertility,” “IVF,” “ART,” “implantation,” “follicular fluid,” “uterine fluid,” and “uterine-luminal fluid,” including both restricted vocabulary and free text terms. Employing Boolean operations and truncation when necessary for the terms “menstrual blood,” “microRNA,” “biomarker,” “endometrial receptivity,” “oocyte quality,” and “embryo development.” The titles and abstracts were assessed for relevance to at least one of three dimensions: follicular/oocyte environment, preimplantation embryo competency, or endometrial receptivity, as well as to at least one non-invasive matrix (serum/plasma, menstrual blood, or uterine/uterine-luminal fluid). Studies analysing peritoneal fluid were included when they clarified the influence of immune-lesion interactions on reproduction.

We included peer-reviewed original research (clinical cohorts, translational studies, and pertinent animal or in vitro embryo models) with exemplary reviews that offered translational or mechanistic insights. Case reports, editorials, conference abstracts without complete texts, and studies without EV characterisation were discarded unless they provided a novel mechanistic feature supported by supplementary sources. We only selected research that conformed to the requirements set out by the International Society for Extracellular Vesicles given that extracellular vesicle analytics are particularly vulnerable to pre-analytical factors. These guidelines encompass the reporting of particle detection (e.g., NTA), morphology (electron microscopy), and protein markers (positive/negative panels via Western blot or bead-based flow cytometry), in addition to isolation specifics and normalisation techniques (exogenous spike-ins and/or endogenous reference miRNAs). In our clinical studies, we documented the cohort design, size, inclusion criteria, disease staging/phenotype, hormonal environment (e.g., COCs, progestins, GnRH analogues), cycle phase, timing of sampling relative to embryo transfer, EV source and isolation technique, cargo modality (miRNA, protein, lipid, metabolite), analytical platform (qPCR, targeted/untargeted NGS, proteomics), and endpoints associated with ART (metaphase-II yield, usable blastocyst rate, morphokinetics, implantation rate, clinical pregnancy, and live birth). When accessible, we obtained effect sizes, calibration metrics, areas under the receiver-operating characteristic curve, and data about internal and external validation.

To guarantee accuracy and standardise language for EV subtypes, sample matrices, and outcome definitions, data extraction was performed twice. We used a qualitative, domain-specific synthesis instead of meta-analysis owing to considerable diversity in research designs and reporting. The evidence was synthesised along a mechanistic-to-clinical continuum by correlating EV cargo with pathways (inflammation, angiogenesis/neurogenesis, fibrosis/ECM, metabolism/oxidative stress, progesterone signalling/epigenetics), associating these pathways with the three reproductive axes, and ultimately assessing biomarker efficacy across matrices while considering pre-analytics and covariates. We focused on research that explicitly connected EV cargo to worse IVF results or intrinsic effects on embryos, such as NOTCH regulation by UF-EV miRNAs. The primary areas of concern regarding bias include selection bias (surgical confirmation versus clinical diagnosis), spectrum bias (lesion phenotype/stage), analytical bias (platelet contamination, haemolysis, blood carryover in UF), and confounding due to hormone usage or cycle dating. To ascertain the reasons for the discrepancies in findings, we examined variations in isolation chemistry (ultracentrifugation, density gradients, SEC, polymer precipitation), normalisation methodologies, and the scheduling of samples concerning the implantation window.

This study did not need informed consent or institutional review board approval, since it examines public data without direct patient participation. The objective of the technique is to synchronise diverse EV evidence with IVF decision-making milestones. The synthesis concludes with a practical framework promoting the use of serum/plasma extracellular vesicles for baseline triage, menstrual-blood extracellular vesicles for non-invasive endotyping, and uterine-fluid extracellular vesicles for pre-transfer receptivity evaluations. It enumerates the necessary procedures for clinical application, including assay calibration, multicentre harmonisation, and decision thresholds.

## 3. Extracellular Vesicles as Molecular Messengers

EVs are lipid-bilayer nanostructures released by almost all cells. They serve as high-fidelity transmitters of molecular information both inside and among tissues. They are altered to promote inflammation, fibrosis, and implantation failure in situations like endometriosis. In reproductive biology, they modulate paracrine and endocrine signalling along the ovarian-tubal-uterine axis [54]. Despite the overlap in biophysics and function across these categories, the EV family is categorised into three groups according to their mechanisms: exosomes (30–150 nm; originating from endosomes), microvesicles (100–1000 nm; formed by outward budding from the plasma membrane), and apoptotic bodies (>1000 nm; produced by dying cells) [55]. All EVs possess an asymmetric phospholipid bilayer abundant in phosphatidylserine, sphingomyelin, and cholesterol. It has distinctive microdomains termed tetraspanin-enriched microdomains that aggregate CD9, CD63, and CD81 to facilitate cargo and direct absorption. The lumen contains miRNAs, lncRNAs, circRNAs, DNA fragments, mRNAs, enzymes, signalling adaptors, and metabolites [56]. The membranes include integrins, heparan-sulphate-binding proteoglycans, and lipid ligands that regulate tissue tropism. Encapsulation stabilises oxidation-sensitive compounds, rendering them resistant to proteases and RNases. This enables EVs to navigate the body and endure severe conditions, such as iron-rich, reactive oxygen species-laden peritoneal fluid [57]. In healthy individuals, reproductive-tract extracellular vesicles facilitate the coordination of follicular development, the contact between gametes and epithelium, and the receptivity of the endometrium. In endometriosis, the same mechanism serves as a conduit for pathological remodelling, relaying inflammatory and pro-angiogenic signals encoded in lesions to immunological, stromal, endothelial, and epithelial targets both locally and distally [58].

The process of EV biogenesis is very sensitive to the metabolic stresses associated with endometriosis and is exceedingly complex. Exosomes are generated as intraluminal vesicles (ILVs) within multivesicular bodies (MVBs) through ESCRT-independent mechanisms that depend on ceramide-rich microdomains (neutral sphingomyelinase 2/SMPD3) and tetraspanin lattices, or via ESCRT-dependent sorting that includes HRS/STAM (ESCRT-0), TSG101 (ESCRT-I), ALIX (ESCRT-associated), and VPS4-mediated scission [59]. The cargo selection is deliberate: lipids and metabolites partition into raft-like domains, ubiquitinated proteins are isolated by ESCRT-0/-I, and miRNAs are integrated via sequence and structural patterns identified by hnRNPA2B1, SYNCRIP/hnRNPQ, YBX1, and AGO2-dependent condensates. Hypoxia (HIF-1α), inflammatory cytokines (TNF/IL-1β), and oestrogenic signalling hallmarks of endometriotic lesions upregulate Rab GTPases (Rab27a/b, Rab11, Rab35) and SNAREs essential for membrane fusion, which are required for MVB trafficking to the plasma membrane [60]. Microvesicles emerge by actomyosin-mediated membrane fission, facilitated by ARF6, RhoA/ROCK, ERM proteins, and scramblase-induced externalisation of phosphatidylserine. Calcium flow, lysophosphatidic acid, and reactive oxygen species accelerate this process. The hyper-secretory EV characteristic of lesion tissue may be explained by the presence of similar stimuli around haemorrhagic endometriomas and inflammatory peritoneum. Endometriosis not only increases the quantity of EVs but also alters their quality by modifying the sorting hubs that select pro-fibrotic proteins (components of the TGF-β-axis), bioactive lipids, and microRNAs (e.g., miR-21, miR-23a, miR-145-5p, and the miR-200 family), collectively forming a persistent disease signature [61].

The molecular composition of extracellular vesicles directly affects recipient cell signalling and reflects the transcriptional, epigenetic, and metabolic conditions of the parent cell. Extracellular vesicle-derived miRNAs (19–25 nt) and long non-coding RNAs/circular RNAs function as post-transcriptional regulators for pathways that influence trophoblast adhesion (ITGβ3/TROPHININ), stromal decidualisation (PR/HOXA10/IGFBP1), angiogenesis (VEGF/ANGPT/TIE2), and folliculogenesis (KITL/KIT, GDF9/BMP15) [62]. The endometriosis-affected eutopic endometrium secretes miR-145-5p and other anti-receptive signals that inhibit NOTCH and impede blastocyst formation. The mid-secretory endometrium in the uterus encapsulates members of the miR-30d and miR-200 families inside extracellular vesicles, altering the adhesion properties, cytoskeletal structure, and metabolic processes of the trophectoderm to enhance the likelihood of implantation [31]. Examples of protein cargo include tetraspanins (CD9/CD63/CD81), integrins (αvβ3, α6β4), ESCRT components (TSG101/ALIX), MMPs/TIMPs, cytokines (TGF-β, IL-6), growth factors (VEGFA, PDGF), and metabolic enzymes (ACSS2). These may facilitate enzymatic activity, scaffold receptor complexes (e.g., LRP1/TGF-β receptors), or transmit signals directly at the membrane (outside-in via integrins) [56]. Lipidomics indicates that sphingolipid and lysophospholipid species engage with the S1P and LPA receptors on stromal and endothelial cells, facilitating cellular movement and the formation of new blood vessels. Significantly, extracellular vesicles often include several payloads, such as miR-23a, which targets SMAD regulators, latent TGF-β, and LOX [63]. This indicates that the same fibrotic network may be targeted again. Even when individual soluble substances are ineffective, extracellular vesicles may nevertheless induce enduring alterations in cellular morphology and behaviour due to their capacity for combination and variation.

The reorganisation of pelvic and systemic compartments in endometriosis depends on the selective absorption and signalling of EVs. Recipient cells internalise extracellular vesicles by phagocytosis, clathrin and caveolin-mediated endocytosis, macropinocytosis, and lipid raft fusion. Phosphatidylserine sensors (TIM/TAM receptors), heparan-sulphate proteoglycans (syndecans/glypicans), and tetraspanin–integrin complexes (e.g., CD9–ITGαvβ3) are essential for docking [64]. NK and CD8+ T lymphocytes diminish their cytolytic activity upon interaction with PD-L1-expressing vesicles or microRNAs that inhibit granzyme and perforin pathways. Macrophages readily internalise extracellular vesicles from lesions and transform into an M2-like, angiogenic, extracellular matrix-remodelling phenotype via the miR-21/miR-146a-mediated inhibition of PTEN/SOCS1 and the activation of STAT3/NF-κB [65]. Exposure to EV-TGF-β and miR-21 induces MMT in the peritoneal mesothelium, reducing its barrier characteristics and promoting lesion invasion. In contrast, endothelial cells react to EV miR-132/miR-126 clusters and VEGF cofactors by activating the PI3K/AKT-eNOS and MAPK/ERK pathways, promoting tube formation and vascular permeability [66]. Elevated levels of miR-145-5p and reduced levels of miR-30d interfere with embryonic adhesion mechanisms, whereas extracellular vesicle-derived members of the miR-200 family modulate cadherin switching and epithelial polarity in the endometrium. The trophectoderm internalises uterine extracellular vesicles via clathrin-mediated and lipid-raft mechanisms. The supplied miRNAs alter NOTCH, Hippo (YAP/TAZ), and WNT/β-catenin pathways, hence influencing cellular compaction, allocation of ICM/TE, and distribution of energy. These alterations result in modifications in morphokinetics and diminished blastulation when the signal is anti-implantation [67].

Hypoxia, iron-induced reactive oxygen species, oestrogen dominance, and chronic cytokinemia together alter the extracellular vesicle circuitry in endometriosis, resulting in a self-perpetuating lesion niche and a deficient uterine milieu [58]. The extracellular vesicles from stromal cells emit fibrogenic signals that activate TGF-β/SMAD2/3 and YAP/TAZ, leading to an increase in COL1A1, FN1, and LOX, hence enhancing tissue stiffness and stabilising adhesions. The extracellular vesicles from lesions include pro-angiogenic microRNAs (miR-21/miR-132), TGF-β effectors, and matrix metalloproteinase inducers that facilitate the formation of new blood vessels and the degradation of the extracellular matrix [68]. Platelet-derived microvesicles provide tissue factor and phosphatidylserine-enriched surfaces that enhance thrombin generation and microthrombi formation, exacerbating local hypoxia and prompting more extracellular vesicle release. Immune-modulating vesicles, conversely, diminish NK cytotoxicity and facilitate macrophage-mediated neuroangiogenesis [69]. Extracellular vesicle microRNAs and enzymes facilitate metabolic reprogramming. miR-4443, linked with extracellular vesicles in menstrual blood, regulates the PI3K/AKT pathway and ACSS2, hence increasing acetyl-CoA levels for SREBP1-mediated lipogenesis and histone acetylation (H3K27ac). This links acetate metabolism to genetic pathways that facilitate growth and motility. Vesicular mtDNA and oxidised lipids activate TLR9 and cGAS-STING, hence intensifying sterile inflammation. These layers together demonstrate how EVs may convert transient harm into chronic sickness and explain why EV signatures effectively indicate both uterine malfunction and lesion activity.

Circulating extracellular vesicles transmit information on the pelvic molecular state into the bloodstream at a systemic level, generating a reservoir of biomarkers that may be readily accessible and correlated with ART outcomes. Serum/plasma tiny EVs include miRNA signatures (such as miR-22-3p, miR-320a, miR-451a, and miR-200b) and protein indicators that monitor inflammatory levels, promote fibrosis, and assess the body’s preparedness for cell acceptance. Integrin patterns may influence luteal perfusion and implantation microhemodynamics by altering the release of Weibel–Palade bodies, the expression of VCAM-1/ICAM-1, permeability, vascular adhesion, and endothelial uptake [70]. Pro-coagulant and NETosis signals from vesicles produced by platelets and neutrophils alter the architecture of the vascular niche. Longitudinal sampling can identify dynamic transitions, from high-inflammatory “red” states indicative of suboptimal MII yield or implantation to “green” receptivity windows characterised by mid-secretory UF-EV miRNA profiles, as EV cargo fluctuates with cycle phase, hormonal treatments (COCs, progestins, GnRH analogues), and stimulation protocols. Notably, UF-ER molecular profile reflects endometrial transcriptomes throughout the implantation window, so revealing a clear mechanistic link between EV biology and IVF results [71]. Multiple studies now associate serum-EV miRNA panels with AUCs over 0.8 for predicting clinical pregnancy and delivery in endometriosis ART cohorts. Table 1 summarises the most commonly reported EV-derived biomarkers and their relationships with oocyte quality, embryo development, implantation, clinical pregnancy, and live birth in women with endometriosis, aiming to translate these cross-compartment findings into clinically relevant signals for IVF decision-making.

An overview of the microRNAs and proteins derived from extracellular vesicles associated with reproductive outcomes in endometriosis patients using ART. Each item includes details on the origin of the EV biofluid, significant molecular constituents, relevant signalling pathways, and associations with ART outcomes such as oocyte competence, embryo quality, implantation rate, CPR, and LBR. The data originates from papers published between 2023 and 2025. EV denotes extracellular vesicle, FF signifies follicular fluid, UF represents uterine fluid, MB indicates menstrual blood, CPR refers to clinical pregnancy rate, LBR stands for live birth rate, and MII pertains to metaphase II oocyte.

Table 1 demonstrates the predictive and mechanistic capabilities of EV cargo in infertility associated with endometriosis across different biological compartments. Uterine-fluid extracellular vesicles (Li et al., 2024; Apostolov et al., 2025) indicate endometrial receptivity and embryo-endometrium communication during the implantation period, while serum and plasma extracellular vesicle miRNAs (Muraoka et al., 2024; Sadati et al., 2024) correlate with systemic metabolic and angiogenic regulation [31,32,51,72]. Follicular-fluid extracellular vesicles (Duval et al., 2024) signify oxidative stress-induced decline in oocyte quality, whereas menstrual-blood extracellular vesicles (Gurung et al., 2025) demonstrate fibrotic and immunological dysregulation [35,37]. Notably, serum EV-based models demonstrated their effectiveness for pre-ART screening by achieving considerable discriminative power (AUC > 0.8) in predicting clinical pregnancy. These results substantiate the notion that extracellular vesicles serve as molecular messengers connecting reproductive capability, local endometrial biology, and systemic inflammation.

## 4. Sources of Extracellular Vesicles in Endometriosis

EVs are dynamic vehicles for molecular communication that traverse all anatomical and metabolic compartments of the reproductive system. Each source in endometriosis reveals a unique aspect of the disease’s biology, with differences in their origin, composition, and function dependent on the isolation matrix [76]. Given that vesicles derived from serum or plasma primarily reflect systemic inflammation and metabolic conditions, vesicles from menstrual blood indicate eutopic and ectopic endometrium, vesicles from peritoneal fluid demonstrate immune lesion interactions, and vesicles from uterine or uterine-luminal fluid facilitate the implantation phase communication between the endometrium and the embryo, comprehending the source-specific landscape of EVs is crucial. These biofluids work together to provide a comprehensive network of non-invasive molecular gateways into infertility linked to endometriosis.

### 4.1. Plasma and Serum Derived EVs: Systemic Mirrors of Local Pathology

EVs produced from plasma and serum provide the most comprehensive and therapeutically accessible insight into the systemic immunological and biochemical anomalies associated with endometriosis compared to other EV sources. These circulating vesicles signify the aggregate cellular activity inside the eutopic endometrium, endometriotic lesions, and the immunological and endothelial compartments that collaboratively contribute to the illness [77]. Thus, the blood extracellular vesicle compartment significantly affects distant reproductive microenvironments, including the ovarian follicle and the endometrium, while also reflecting local disease.

From a pathogenic perspective, the systemic extracellular vesicle landscape in endometriosis represents an interplay among many organs, originating in the peritoneal cavity and disseminating throughout the circulation. Endometriotic lesions exhibit substantial activation of HIF-1α, NF-κB, and STAT3 as a consequence of cyclical hypoxia, oxidative stress, and iron overload stemming from repeated haemorrhage, all of which are powerful inducers of extracellular vesicle biogenesis [78]. This molecular stress may increase vesicle secretion rates twofold by activating the Rab27a/b, TSG101, and nSMase2 (SMPD3) pathways relative to healthy controls. Cells from lesions, including stromal and epithelial, secrete exosomes abundant in integrins (αvβ3, α6β4) and tetraspanins (CD9, CD63, and CD81) [79]. These exosomes facilitate cellular adhesion to immunological and endothelial targets. Upon their release into the circulation, these vesicles amalgamate with those produced by endometrial cells, monocytes, and activated platelets, forming a complex vesicular milieu that disseminates disease-associated molecular cargo throughout the body.

The molecular contents of circulating extracellular vesicles may provide a comprehensive overview of the inflammatory and metabolic milieu inside the body. Proteomic and transcriptome profiling studies consistently demonstrate that inflammatory mediators such as S100A8/A9 (calprotectin), complement components (C3, C4b, factor B), cytokines (IL-6, TNF), and pro-angiogenic proteins (VEGF-A, ANGPTL4) are present in greater abundance [80]. To promote pro-survival and pro-proliferative conditions, several chemicals are accompanied by signalling adaptors that initiate the PI3K/AKT, JAK/STAT, and MAPK/ERK pathways in the recipient cells. Lipidomic investigations reveal elevated levels of lysophosphatidylcholine and sphingosine-1-phosphate in these EVs. These metabolites are recognised for their role in promoting angiogenesis and enhancing vascular permeability, which serve as mechanistic markers of the proliferation and spread of endometriotic lesions [81].

A particularly stable and useful group of biomarkers is the miRNA content of EVs derived from serum and plasma. Multiple independent cohorts indicate that women with endometriosis have a specific pattern of EV-miRNA dysregulation. Three vesicular miRNAs, miR-22-3p, miR-320a, and miR-200b-3p, which regulate oxidative stress and the EMT, exhibit considerable alterations in circulation and are associated with illness severity and IVF results [82]. Elevated levels of miR-21-5p and miR-146a-5p, which are traditional responders to NF-κB activation, promote macrophage M2 polarisation and inhibit NK-cell cytotoxicity, hence enhancing systemic immunological tolerance to ectopic implants. In contrast, persistent fibrotic remodelling is facilitated by the downregulation of let-7b-3p and miR-451a, recognised as anti-fibrotic and anti-proliferative regulators [83]. A considerable proportion of these dysregulated miRNAs have shared targets in the oocyte competence and endometrial receptivity pathways (e.g., PTEN, SMAD4, ESR1), thereby connecting the molecular disease identified in blood to certain reproductive outcomes.

Circulating EVs function as vectors disseminating disease biology outside the pelvis, rather than only serving as passive reflections. Upon the uptake of EVs from lesions by endothelial cells, eNOS and VEGF receptor 2 are activated, resulting in angiogenesis in distant regions of the body. Similarly, endometrial EV exposure in hepatic or adipose tissue alters lipid metabolism via ACSS2, exacerbating the patients’ low-grade systemic metabolic inflammation [39]. Vesicular cargo in immune cells suppresses cytotoxic NK cell responses and modifies the development routes from monocytes to macrophages, creating an immunologically tolerant environment that promotes lesion persistence. The persistent effects demonstrate the activity of EVs as endocrine-like mediators connecting localised inflammation to systemic immunological and metabolic dysfunction [84]. EVs in serum and plasma have attracted interest as non-invasive indicators for predicting the efficacy of ART in women with endometriosis. Muraoka et al. (2024) revealed in a prospective IVF cohort that two serum extracellular vesicle miRNAs, miR-22-3p and miR-320a, outperformed traditional indicators like serum oestradiol and endometrial thickness in forecasting clinical pregnancy and live birth, attaining an area under the ROC curve (AUC) greater than 0.8 [51]. The cumulus-oocyte complex and endometrial biopsies demonstrated transcriptomic changes indicated by these EVs, suggesting that systemic vesicles assimilate signals from the whole reproductive axis. Brady et al. (2024) discovered that the concurrent expression of five plasma extracellular vesicle microRNAs (miR-542-3p, let-7b-3p, miR-548i, miR-769-5p, and miR-30c-1-3p) proved beneficial for diagnosis, even in the initial phases of the illness [73]. The AUC for distinguishing between teenagers with surgically proven endometriosis and healthy controls was 0.77.

Circulating extracellular vesicles provide insights regarding treatment efficacy and diagnostic forecasting. Following gonadotropin stimulation, GnRH analogue therapy, and laparoscopic lesion removal, changes in serum ER molecular profiles have been seen, indicating their potential as dynamic biomarkers for treatment assessment [85]. Baseline triage before to stimulation, mid-cycle assessment of ovarian response, and luteal-phase evaluation of receptivity represent three IVF checkpoints where EV profiling might be included, given that blood collection is less intrusive and conducive to repeated sampling. Combining serum-EV data with hormonal and metabolic factors may provide multivariate algorithms that much exceed single-analyte assays in forecasting oocyte yield, embryo quality, or implantation likelihood [86].

Serum and plasma extracellular vesicles at the molecular pathologic level elucidate the dissemination of endometriosis biology throughout the organism. This represents an axis of oxidative stress, chronic inflammation, and immunological dysregulation that impedes follicle formation and endometrial receptivity [87]. The oxidative load of the illness is shown by the vesicular encapsulation of redox enzymes (peroxiredoxin 3, SOD2), mitochondrial fragments, and oxidised phospholipids. Simultaneously, concurrent miRNAs that inhibit antioxidant defences exacerbate ROS-induced DNA damage in reproductive and peripheral organs [88]. This circulating oxidative fingerprint connects systemic metabolic stress and reproductive inefficiency with pelvic disease. Plasma and serum extracellular vesicles constitute the “hologram” of endometriosis. Their composition precisely represents the inflammatory, fibrotic, and metabolic pathways inherent in the lesions. Thus, they serve as a mechanistic link between the molecular pathophysiology of endometriosis and its clinical manifestation in infertility, while also acting as an accessible diagnostic tool.

### 4.2. Menstrual-Blood-Derived EVs: A Direct and Non-Invasive Window into Endometrial Biology

A non-invasive and instructive biopsy of the eutopic endometrium, and to a lesser degree, exfoliated ectopic foci, may be obtained from menstrual blood during a time of substantial tissue remodelling [89]. Every phase of menstruation is abundant in extracellular vesicles and encompasses regulated inflammation, extracellular matrix degradation, haemostasis, and the re-epithelialization of stromal and epithelial cells. The principal sources of MB-EVs include endometrial epithelial cells, stromal fibroblasts, and infiltrating immune cells, such as neutrophils, macrophages, and uterine NK cells [7]. Platelets and endothelial cells exposed to localised hypoxia and iron-induced oxidative stress also contribute. This environment promotes the synthesis of exosomes and microvesicles via the ESCRT (TSG101/ALIX), nSMase2/ceramide, and Rab27a/b pathways by upregulating HIF-1α, NF-κB, and prostaglandin signalling [90]. The vesicular membranes, abundant in CD9/CD63/CD81 and certain integrins, effectively record the endometrium’s changes from the proliferative to secretory to menstrual phases. These membranes include miRNAs, lncRNAs/circRNAs, mRNAs, proteins, lipids, and metabolites. Certain MB-EVs may originate from ectopic deposits and immune cells inside the peritoneum due to retrograde flow occurring concurrently with menstruation [91]. This enables a “blended” signature of eutopic ectopic interaction without the need for surgical intervention. MB-EV cargo is quantitatively and qualitatively altered to enhance pro-fibrotic, pro-migratory, and proliferative signalling in women with endometriosis. Multiple investigations have shown increased vesicular levels of miR-21-5p, miR-23a, miR-29c, and miR-4443. These components converge on PI3K/AKT–mTOR and ACSS2-mediated acetate metabolism [92]. MiR-4443 establishes a mechanistic link between metabolic flux and chromatin accessibility at genes regulating migration, proliferation, and extracellular matrix synthesis by enhancing ACSS2 activity and replenishing acetyl-CoA to facilitate SREBP1-mediated lipogenesis and histone acetylation (e.g., H3K27ac) [93]. Naïve endometrial stromal cells exposed to MB-EVs from afflicted individuals exhibit elevated levels of COL1A1, FN1, α-SMA, and LOX. These indicate myofibroblast transformation and matrix stiffness via TGF-β/SMAD2/3 and YAP/TAZ–TEAD activation. Targeting PTEN/RhoGDI, concurrent EV-derived miRNAs augment RhoA/ROCK activity, promote stress fibre and focal adhesion (FAK/Src) creation, hence enabling invasion and compromising the mesothelial barrier—cellular mechanisms essential for adhesion formation and lesion implantation [94].

In addition to fibrosis, MB-EVs transport neuroangiogenic and immunological mediators that alter the peritoneal milieu. Proteomic analyses of tiny extracellular vesicles in menstrual fluid (>5000 proteins identified) reveal synchronised alterations in innate immunity (S100A8/A9, complement), coagulation (fibrinogen chains, TF-bearing microvesicles), angiogenesis (VEGF-axis co-factors), and cytoskeletal metabolism [35]. Cases of endometriosis have a relative deficiency of cytodifferentiation enzymes and antioxidant defences (SOD2/PRDXs), correlating with elevated oxidative stress. Mesothelial cells undergo mesothelial-to-mesenchymal transition leading to the dissociation of junctional proteins and the promotion of ectopic adhesion. MB-EVs also prime macrophages to adopt an M2-like phenotype and elevate levels of CD86 and other activation markers [95]. Neurotrophic signals, in conjunction with miRNAs that affect the semaphorin/neuropilin and netrin guidance pathways, result in aberrant innervation and neuro-immune synapses that induce dysmenorrhea and dyspareunia. Conversely, vesicular S1P/LPA species activate GPCRs to promote endothelial sprouting and permeability. By further stimulating TLR9 and cGAS-STING, EV-encapsulated mtDNA and oxidised lipids may reinforce nociceptive sensitisation and sterile inflammation [96].

MB-EVs serve as a substantial source for diagnosis and long-term surveillance due to their origin from both exfoliated ectopic tissue and eutopic endometrium. Time-resolved profiling with low patient burden is enabled by the ease of collection techniques (menstrual cups, pad/napkin eluates) and their consistency across cycles [97]. In reality, MB sampling on cycle days 1–3 produces reliable vesicle counts, repeatability is enhanced by following MISEV-compliant pre-analytics (rapid chilling, platelet depletion, haemolysis assessments) and orthogonal characterisation (NTA/EM, Western blotting, or bead-based flow for CD9/CD63/CD81/TSG101). Analytical pipelines must account for factors affecting EV cargo, such as blood cell carryover, cycle day, hormonal exposure (combined oral contraceptives, progestins, and GnRH analogues), and pain severity. MB-EV panels may forecast IVF readiness, monitor therapeutic responses (both medicinal and surgical), and categorise patients along inflammatory, fibrotic, and neuroangiogenic axes by indicating cyclic endometrial fitness before stimulation or transfer [98].

By stabilising cycle-coupled endometrial biology, MB-EVs enhance serum and uterine fluid metrics within the framework of ART. In conjunction with ECM-remodelling proteins, elevated levels of vesicular miR-4443/miR-21-5p may assist in identifying individuals at risk for an adverse oocyte environment (due to systemic inflammation) and difficulties with implantation [32]. This information may assist you in determining whether to optimise pre-treatment, customise stimulation, or freeze everything when the MB-EV signature indicates a hostile peri-implantation condition. In contrast, the reestablishment of a “healthy” MB-EV profile, defined by reduced fibrotic and oxidative markers together with a restored miR-30d/miR-200 equilibrium, may indicate improved receptivity and enable a fresh transfer. MenSC-derived EVs are under investigation as therapeutic agents due to their immunomodulatory and anti-fibrotic properties that may alter the endometrial microenvironment. This indicates that the same biofluid may provide both diagnostic and therapeutic solutions. MB-EVs provide direct, physiologically relevant molecular insights on endometrium and lesion dynamics, together with the scalability and patient acceptance necessary for integrating EV biology into regular reproductive treatment.

### 4.3. Peritoneal-Fluid-Derived EVs: Immune–Lesion Crosstalk and Inflammatory Amplification

The peritoneal cavity is the centre of the pathophysiology of endometriosis. It is a dynamic space where immune cells, mesothelial layers, and ectopic endometrial lesions interact in a way that is always inflammatory and fibrogenic [99]. PF-EVs serve as highly active messengers among cells within this microenvironment, orchestrating the molecular interactions that sustain lesion implantation, proliferation, angiogenesis, and immunological tolerance. These vesicles, originating from neutrophils, macrophages, endothelial cells, fibroblasts, and ectopic endometrial cells, encapsulate the molecular essence of endometriosis pathophysiology and signify the immunometabolic state of the peritoneal environment.

PF-EVs are common at the cellular level because the peritoneal microenvironment has cyclical bleeding, oxidative damage, and constant mechanical stress. Retrograde menstruation brings haemoglobin, iron, and apoptotic debris to mesothelial cells and macrophages. This process starts lipid peroxidation and turns on the cGAS–STING and TLR2/4–NF-κB pathways [100]. These signals, the activation of ESCRT machinery by oestradiol, and Rab27a/b-mediated exocytosis all lead to a hypersecretory vesicular phenotype. The PF of women with endometriosis has been shown to have much higher levels of small EVs (50–150 nm) that are rich in tetraspanins (CD9, CD63, and CD81) and adhesion molecules like integrin αvβ3 and ICAM-1 [101]. These molecules help the cells stick to stromal fibroblasts and mesothelial surfaces, as shown by electron microscopy and nanoparticle tracking analysis. These vesicles change the peritoneal niche to make ectopic implantation easier. They also act as biochemical messengers and micro-scale effectors.

The molecular cargo of PF-EVs illustrates their essential role in immune modulation and lesion preservation. Transcriptomic analyses show that miR-21, miR-23a, miR-214, miR-451a, and miR-142-3p are all more common. These miRNAs work together to stop apoptosis, speed up angiogenesis, and start fibroblast activation. These miRNAs target key regulators such as PTEN, SMAD7, and TIMP3 to turn on the PI3K/AKT, TGF-β/SMAD, and EMT-like pathways [102]. These networks help ectopic stromal cells stay alive, help the immune system avoid detection, and make it easier for cells to move around. Proteomic analysis of PF-EVs reveals an over-representation of MMP-2, MMP-9, VEGF-A, FGF2, IL-6, and S100A8/A9 molecules that collectively facilitate matrix degradation, angiogenesis, and sustained inflammatory signalling. Additionally, vesicle-bound oxidised phospholipids and sphingolipids enhance the inflammatory response by binding to macrophage TLR4 and CD14 receptors, sustaining NF-κB activation and the secretion of TNF-α and IL-1β [103].

One of the things that makes PF-EVs stand out is that they can change macrophages, which are the main type of immune cell in peritoneal fluid, into an M2-like, pro-lesion but pro-repair phenotype. Endometriosis-derived PF-EVs divert macrophages from classical (M1) activation by delivering miR-21-5p, miR-146a-5p, and let-7d-5p, which inhibit transcription factors such as IRF5 and STAT1 [104]. The final result is a group of cells that makes cytokines like IL-10, TGF-β, and CCL18 that stop cytotoxic lymphocytes and promote angiogenesis and fibrosis. PF-EVs reduce NK-cell cytotoxicity and make it harder to find and kill ectopic endometrial cells at the same time by sending miR-214 and PD-L1-enriched exosomes. These immune evasive effects let endometriosis last a long time by making lesions last longer and lowering immune clearance [105].

PF-EVs not only change the immune system, but they also directly help angiogenesis and neurogenesis, which are two processes that are closely linked to pain and the growth of lesions. In EV cargo, there are miRNAs that stop anti-angiogenic regulators like PTEN and THBS1 from working [106]. There are also VEGF-A, ANGPT1, and neuropilin-1. Vesicular neurotrophic factors such as BDNF and NGF promote sensory nerve outgrowth associated with specific pelvic pain syndromes. Concurrently, the uptake of these vesicles by endothelial cells activates eNOS and ERK1/2, facilitating sprouting and capillary formation. Recent single-EV sequencing has revealed the co-packaging of angiogenic and neurogenic mRNAs, suggesting that PF-EVs function as molecular multipliers, facilitating both innervation and vascularization within lesions concurrently [107].

The role of PF-EVs in lesion adhesion and the disruption of the mesothelial barrier is a significant mechanistic aspect. Vesicles from ectopic endometrial cells and activated macrophages cause MMT by sending signals that activate Snail/Slug and TGF-β1 and miR-200b [108]. This leads to the upregulation of vimentin and fibronectin and the downregulation of E-cadherin. This change makes the mesothelium weaker, shows the basement membrane underneath, and makes it easier for endometrial fragments that have flowed back to implant. At the same time, TF and matrix metalloproteinases (MMP-2, MMP-9) in PF-EVs help with local fibrin deposition and proteolysis, which helps with adhesion stability and angiogenic sprouting at the edge of the lesion [109].

The oxidative environment of the peritoneal cavity changes the makeup of PF-EV even more, making them more likely to cause disease. Cyclic bleeding causes high levels of iron, which start Fenton chemistry and make hydroxyl radicals that oxidise proteins and phospholipids on vesicular membranes [110]. To keep sterile inflammation going, these changes make neoepitopes that stick to pattern recognition receptors like TLR4 and RAGE. Vesicular oxidised low-density lipoprotein fragments have been identified as potent macrophage activators, linking immune dysregulation and lipid peroxidation. Oxidative stress also encourages the inclusion of peroxiredoxin 2 and HSP70/90 into PF-EVs. Both of these proteins act as DAMPs that keep inflammation and pain sensitivity going through TRPV1 and NF-κB signalling [111].

PF-EVs are a useful clinical source of diagnostic and mechanistic data, even though accessing them is intrusive. High-throughput sequencing has effectively characterised PF-EVs acquired via laparoscopy, and ongoing research consistently reveals distinct EV-miRNA and proteomic signatures differentiating patients with endometriosis from control subjects. Sadati et al. (2024) identified plasma and peritoneal EV miRNAs (miR-451a, miR-148a, miR-23b, miR-100, and miR-154) that effectively differentiate endometriosis, demonstrating the translational potential of PF-derived signatures [72]. This matrix remains highly beneficial for pathophysiological exploration, biomarker validation, and the identification of therapeutic targets influencing the peritoneal inflammatory network, despite the impracticality of routine PF sampling in clinical IVF settings.

### 4.4. Uterine-Fluid and Uterine-Luminal EVs: The Molecular Interface of Implantation

The uterine cavity is the last place where reproduction can either succeed or fail. During the mid-secretory phase, also known as the WOI, the endometrium undergoes a carefully planned change in response to progesterone, oestradiol, cytokines, and growth factors [112]. This change prepares the endometrium for the arrival of the embryo. Uterine-fluid and uterine-luminal extracellular vesicles have emerged as significant molecular intermediaries facilitating communication between the endometrium and the preimplantation embryo within this temporally delineated microenvironment. These vesicles are like molecular translators that package and send lipid, transcriptomic, proteomic, and epigenetic signals. They make sure that the mother’s body is ready to receive the embryo and that the embryo is ready to develop [113].

Decidual macrophages, uterine uNK cells, endometrial epithelial and stromal cells, together with infiltrating lymphocytes and endometrial endothelium cells, release UF-/ULF-EVs. Their production varies cyclically owing to the synergistic influences of progesterone and local paracrine hormones, such as VEGF, LIF, and IL-11, with a notable rise during the mid-secretory phase [114]. These hormone and cytokine signals facilitate exosome secretion and selective cargo loading, while concurrently activating pathways reliant on Rab27a/b, ALIX, and ESCRT-III. Recent multi-omics analysis indicates that the molecular composition of UF-EVs resembles the gene expression in endometrial tissue over the menstrual cycle [32]. The research revealed significant concordance in the signatures of mRNA, miRNA, and surface proteins. As the cycle progresses, the miRNA and mRNA profiles inside UF-EVs shift from proliferative (cell cycle, WNT/β-catenin-regulated genes) to secretory (decidualisation and adhesion-related transcripts) states [115]. The UF-EV surface proteome, abundant in CD56, CD45, CD3, and CD142 during the mid-secretory phase, exhibits a coordinated immune-epithelial remodelling that promotes regulated inflammation, angiogenesis, and immunological tolerance. All of these factors are essential for successful implantation.

The miRNA composition of UF-/ULF-EVs provides a thorough molecular depiction of endometrial receptivity. The most recognised are miR-30d-5p, miR-200b-3p, miR-200a-3p, and miR-141-3p. They regulate adhesion and invasion mechanisms by modulating ITGβ3, osteopontin (SPP1), LIF, and MUC1 [116]. These vesicular miRNAs function locally and are absorbed by preimplantation embryos, thus altering the trophectoderm transcriptomes and enhancing the activity of adhesion molecules. In vitro models of humans and mice exhibit improved blastocyst attachment and hatching when subjected to UF-EVs from the receptive phase, indicating that vesicle transfer functions as a physiological mechanism for maternal–embryonic signalling. If this miRNA cargo is malfunctioning, women with endometriosis are unable to achieve implantation. Li et al. (2024) revealed that UF-EVs from endometriosis patients have increased levels of miR-145-5p, which inhibits NOTCH1/2 signalling in both murine and human embryos, leading to reduced implantation rates and inadequate blastocyst development [31]. This research elucidates a molecular link between altered endometrial extracellular vesicle cargo and developmental abnormalities seen in embryos.

The protein composition of UF-EVs highlights their crucial role in the molecular dialogue of implantation. Vesicular proteins include tetraspanins (CD9, CD63, and CD81) that stabilise and internalise vesicles, cytoskeletal regulators (actin, annexins, and ezrin) that modulate cellular adhesion, and immune and coagulation mediators, such as complement components, HLA-G, and CD142 (tissue factor), which impact local immune tolerance [117]. In the WOI, osteopontin, galectins, and integrins (αvβ3, α6β4) are present in significant quantities in UF-EVs. These proteins facilitate the adhesion of the blastocyst to the luminal epithelium. Proteomic analyses of infertile and fertile women indicate that UF-EVs from endometriosis patients demonstrate downregulation of adhesion-related and antioxidant proteins (e.g., PRDXs, GPXs) with increase in pro-coagulant and inflammatory factors [118]. This signifies a shift to a hypoxic, pro-oxidative, and immune-activated uterine environment that negatively impacts implantation.

The UF-EV molecular signature serves as a non-invasive marker of implantation readiness, reflecting the receptivity status of the endometrium. The responsive endometrial phenotype results from three processes facilitated by vesicles accumulated during the mid-secretory phase in normal cycles: trophectoderm differentiation, epithelial junctional remodelling, and stromal decidualisation. The dysregulation of vesicular miRNAs, including miR-30d, miR-200b, and miR-148b, results in a “silent” endometrium incapable of responding to embryonic cues [119]. High-throughput sequencing of endometrial fluid has identified predictive miRNA models that can differentiate between implantative and non-implantative endometria, achieving AUCs exceeding 0.9 in discovery cohorts and surpassing 0.75 in independent validation [116]. The results indicate that the molecular signature of receptivity is linked to EVs rather than free-circulating microRNAs. Preimplantation embryos release EVs that carry miRNAs, including miR-661 and miR-372, which may alter endometrial gene expression to promote adhesion and invasion. Furthermore, EV-mediated crosstalk is bidirectional. To enhance implantation effectiveness, maternal and embryonic EVs perpetually modify their molecular programmes via reciprocal communication, therefore creating a vesicle-based feedback loop [120]. When endometrial inflammation or endometriosis alters the composition or quantity of extracellular vesicle cargo, the synchronisation between the embryo and endometrium is disrupted. This results in unsuccessful implantation or early pregnancy termination.

The characterisation of UF-/ULF-EVs has exceptional translational potential for reproductive medicine. In contrast to invasive biopsies, uterine-fluid sampling enables real-time assessment of receptivity and may be conducted during mock cycles or just before embryo transfer [32]. Due to its examination of both static gene expression and dynamic post-transcriptional and intercellular communication, EV analysis may surpass existing transcriptomic assays, such as ERA. Pilot IVF studies confirm the use of UF-EV miRNA profiles as functional biomarkers by illustrating their association with implantation and pregnancy outcomes, specifically the ratios of miR-30d to miR-200b [121]. Furthermore, monitoring UF-EV signatures may improve decision-making about endometrial priming, transfer time, and the need for supplementary therapy, such antioxidant or anti-inflammatory drugs. Therapeutic prospects are starting to emerge beyond diagnostics. Engineered EVs generated from healthy endometrial epithelial or mesenchymal stem cells may enhance receptivity by transmitting correcting miRNAs or proteins, such as LIF, VEGF, or PRDX2. Menstrual blood-derived MenSC-EVs have shown the ability to reduce oxidative stress and fibrosis, indicating their potential as a therapeutic approach for endometrial dysfunction resulting from endometriosis. The integration of UF-EV profiling with serum and menstrual-blood EV data might facilitate the development of multi-compartment biomarker panels that examine the whole reproductive axis, from follicle to implantation, as a singular molecular continuum [122].

### 4.5. Integrative Perspective on EV Sources: From Pathophysiology to Translational Biomarkers

The many sources of EVs serum/plasma, menstrual blood, peritoneal fluid, and uterine fluid offer distinct but interconnected molecular insights inside the reproductive system. Each matrix delineates a distinct aspect of infertility linked to endometriosis: implantation-phase endometrial embryo communication (uterine-fluid EVs), cyclic endometrial remodelling and lesion shedding (menstrual-blood EVs), local immune–lesion interaction and inflammatory amplification (peritoneal-fluid EVs), and systemic inflammation and immune dysregulation (serum/plasma EVs). These vesicular populations cooperate to create a multi-compartment signalling network that includes events ranging from implantation failure to lesion development. An extensive foundation for mechanistic knowledge and translational application is provided by the grasp of the interconnections among these vesicular streams.

#### 4.5.1. Molecular Continuum: Connecting Systemic and Local Vesicular Pathways

The EV system in endometriosis functions as an integrated whole rather than as distinct compartments. Rather, it functions as a bidirectional communication network. EVs originating from lesions, discharged into the peritoneal cavity, may traverse the peritoneal barrier and infiltrate the circulation [37]. These EVs transport pro-inflammatory and pro-angiogenic substances that may influence the immune system, liver, and ovaries. In serum EVs, the same peritoneal signatures comprising miR-21-5p, miR-23a, VEGF-A, and TGF-β1 reappear and function as systemic enhancers of localised disease [123]. EVs produced from menstrual blood may fuse with circulating vesicles to propagate metabolic and fibrotic signals throughout the body. These EVs represent both eutopic and ectopic endometrial transcriptional profiles. In contrast, systemic extracellular vesicles originating from endothelium or activated immune cells return to the reproductive tract, modifying the recruitment of immune cells in the ovary and endometrium, along with the tone of the microvasculature. The metabolic and inflammatory manifestations of endometriosis, such as fatigue, dyslipidaemia, and disrupted oxidative balance, can be explained by the molecular loop created by ongoing vesicular exchange, linking pelvic pathology to broader physiological processes [124].

The integration of vesicles is facilitated by common molecular pathways, including NF-κB, PI3K/AKT/mTOR, and TGF-β/SMAD, which regulate EV synthesis, cargo selection, and tissue uptake. Progesterone resistance and oestrogen dominance alter the miRNA and protein concentrations in extracellular vesicles secreted by endometrial cells. Conversely, oxidative and inflammatory stress in lesions activates Rab27a/b and nSMase2, facilitating the release of vesicles. Upon traversing the body, certain specific miRNAs (such as miR-21, miR-23a, and the miR-200 family) alter the transcriptional networks of immune cells, resulting in angiogenesis and chronic inflammation [125]. The final result is a feed-forward loop of vesicle-mediated signalling, whereby systemic inflammation conditions the uterus for diminished receptivity and compromised implantation, while local lesion activity influences systemic biofluids.

#### 4.5.2. Functional Crosstalk: Effects on the Endometrial, Follicular, and Ovarian Compartments

Peritoneal and systemic inflammation are linked to altered oocyte competency via vesicular communication that permeates the ovarian and follicular environment. Granulosa and cumulus cells may absorb extracellular vesicles from serum or peritoneal sources, altering their programming by inhibiting PTEN and activating PI3K/AKT. This results in premature luteinisation, mitochondrial dysfunction, and a reduced quantity of metaphase-II oocytes [126]. Simultaneously, EVs abundant in S100 proteins, complement fragments, and oxidised lipids induce apoptosis and endoplasmic reticulum stress in granulosa cells, jeopardising follicular homeostasis. The data demonstrate the correlation between follicular ageing and systemic inflammation, linking the extragonadal disease load of endometriosis to reduced gamete quality [37].

At the uterine level, UF-EVs represent the last stage in this sequence. They serve as functional interpreters of all upstream molecular disturbances: endometrial epithelial and stromal cells release extracellular vesicle cargo, where oxidative imbalance, immunological activation, and hormonal dysregulation occur. Consequently, in UF-EVs originating from endometriosis patients, anti-receptive miRNAs such as miR-145-5p and miR-155-5p are elevated, whereas the miR-30d/miR-200b/LIF/STAT3 signalling axis, crucial for receptivity, is often downregulated [52]. These alterations result in suboptimal implantation outcomes and inadequate communication between the trophectoderm and the endometrium during in vitro fertilisation. Reproductive failure is shown to be a direct phenotypic expression of vesicle-mediated disease rather than a simple downstream effect, since the same EV-driven molecular processes that enable lesion persistence also exhibit as functional infertility.

#### 4.5.3. Integrating Diagnostics to Formulate a Multi-Matrix Vesicular Signature

The amalgamation of their fingerprints holds significant promise for identifying multi-dimensional biomarkers, since extracellular vesicles traverse compartments freely and possess partly overlapping contents. A future diagnostic paradigm might integrate serum EV miRNAs (indicative of systemic inflammation), menstrual-blood EV markers (reflecting lesion activity and cycle repair), and UF-EV miRNA/protein profiles (for receptivity evaluation) into a singular prediction model [127]. This composite biomarker would provide a precise diagnosis of endometriosis while simultaneously assessing reproductive potential and ART prognosis. Initial modelling suggests that multi-source EV panels surpass single-fluid tests in differentiating between implantative and non-implantative endometria. When accurately calibrated for pre-analytical variability, they may achieve AUC values beyond 0.9.

To implement this integrative approach, adherence to Minimal Information for Studies of Extracellular Vesicles guidelines is crucial to ensure reproducibility across normalisation strategies (particle counts, protein load, or housekeeping miRNAs) and isolation platforms (ultracentrifugation, size-exclusion chromatography, and precipitation kits) [128]. Artificial intelligence and machine learning methods may enhance diagnostic accuracy by integrating molecular profiles from extracellular vesicles with hormonal, metabolic, and imaging data. This will provide a systems-level fertility index for customised IVF therapy.

#### 4.5.4. Clinical and Therapeutic Implications of Translational Importance

The thorough examination of EV sources offers insights into therapeutic intervention opportunities as well as their diagnostic functions. Inhibiting vesicle release or uptake routes, such as via nSMase2 inhibitors (GW4869), Rab27 inhibition, or neutralising antibodies targeting integrins and tetraspanins, may disrupt the EV-mediated inflammatory cycle [129]. This would prevent the lesions from enlarging and render them responsive once again. Therapeutic extracellular vesicles produced from mesenchymal stem cells, endometrial epithelial cells, or menstrual stromal cells may convey anti-fibrotic microRNAs (miR-29 family), anti-inflammatory cytokine mimetics (IL-10), or angiogenesis regulators (VEGF, PRDX2). In preclinical models, exogenous EVs carrying miR-223 or miR-125b have shown improved implantation outcomes, decreased peritoneal inflammation, and reprogrammed macrophages to assume homeostatic phenotypes [130].

The ability of multi-source EV research to convert endometriosis from a surgically confirmed illness into a molecularly stratified condition suitable for non-invasive diagnosis and monitoring exemplifies its critical translational importance. By delineating the vesicular dialogue across tissues, researchers may identify individuals predisposed to react to certain treatments, such as hormonal therapy, anti-inflammatory medications, or precisely timed IVF, and thereafter monitor their molecular recovery over time. Incorporating EV-based indices into ART processes might render vesicular biology a valuable clinical intelligence tool, facilitating customised ovarian stimulation, cycle readiness assessment, and implantation scheduling algorithms.

## 5. Molecular Content and Pathogenic Roles of Extracellular Vesicles in Endometriosis

EVs are not only byproducts of cellular apoptosis and regeneration; they serve as active transmitters of pathological information. The primary biochemical indicators of endometriosis are chronic inflammation, oxidative stress, angiogenesis, immunological dysregulation, and fibrosis. These indicators are conveyed and transmitted via miRNAs, mRNAs, proteins, lipids, and metabolites [131]. EVs effectively convert endometriosis into a multicellular signalling disorder, where the disease’s spread depends equally on intercellular communication and the proliferation of localised lesions, acting as cell-free messengers that may modify recipient cell activity. The molecular composition of these vesicles illustrates the impact of endometriosis on the ovarian, peritoneal, and endometrial microenvironments, eventually influencing reproductive success [132]. Furthermore, these activities are temporally controlled by EVs: their synthesis is affected by hypoxia, oestradiol, prostaglandins, and iron-catalysed reactive ROS, while their absorption is limited by the composition of target cell membranes and receptor availability. This results in notable messaging that corresponds with ART therapies and the menstrual cycle. Due to this encoding of time and place, EVs do more than just replicate illness; they orchestrate it. They do this by assembling programmed combinations of regulators that collectively target the same pathway at several regulatory nodes. This induces enduring alterations in phenotype at minimal dosages and ensures that pathological conditions such as neuroangiogenesis, progesterone resistance, and fibrotic locking persist over cycles, even when upstream stimuli fluctuate [133].

### 5.1. Oxidative Stress and Redox Imbalance Indicating

Oxidative stress serves as a crucial biochemical indicator of endometriosis and significantly influences the production and composition of EVs. The Fenton reaction induces the degradation of endometriotic cysts, resulting in the repeated deposition of iron. This generates many ROS, which damage mitochondria, carbonylate proteins, and oxidise lipids. In this environment, lesions and immune cells secrete EVs abundant in mitochondrial DNA, peroxiredoxin fragments, and oxidised phospholipids [134]. These molecules function as damage-associated molecular patterns. These EVs prolong sterile inflammation by activating the TLR4, NLRP3, and cGAS–STING pathways in mesothelial cells and macrophages. The vesicular membrane acts as a redox amplifier upon the oxidation of cardiolipin and phosphatidylserine, resulting in increased curvature and enhanced fusogenic capacity. Aldehyde-adducted proteins inside EVs concurrently evade proteasomal degradation, therefore prolonging their signalling half-life in recipient cells [135].

The adaptive response to oxidative stress is also seen in extracellular vesicle cargo. Vesicular miRNAs, including miR-21-5p, miR-23a-3p, and miR-146a-5p, target antioxidative regulators such as SOD2, GPX4, and NRF2, therefore undermining the antioxidant defences of recipient cells and exacerbating redox imbalance. Proteomic investigations indicate that heat-shock proteins (HSP70/90), ferritin light chain, and peroxiredoxin 2 are abundantly present [136]. These proteins collaborate to regulate iron metabolism and stress signalling. The uptake of these vesicles by granulosa or endometrial cells results in mitochondrial depolarisation, ATP depletion, and increased generation of ROS. These factors impede egg maturation and hinder uterine receptivity for conception. Besides mitochondria, EVs transport ACSS2 and mechanisms that elevate acetyl-CoA concentrations. These processes link oxidative stress with histone acetylation at stress-responsive sites, maintaining a non-adaptive transcriptional state. They emit signals that activate PARP, use NAD+, and disrupt redox buffering (GSH/GSSG).

Serum extracellular vesicles from patients with endometriosis have a unique oxidative profile at the systemic level, marked by increased oxidised cardiolipin, nitrosylated proteins, and lipid peroxidation products [137]. These vesicles affect distant organs, including the ovary and endometrium, by transmitting and propagating the oxidative burden from the pelvic cavity. Oxidative stress generates extracellular vesicles, which subsequently disseminate oxidative damage. This establishes a feed-forward oxidative cycle that connects systemic reproductive failure to localised iron excess. Redox-skewed extracellular vesicles reduce detoxifying efficacy during the crucial shift to receptivity by reducing NRF2/KEAP1 signalling in stromal cells and decreasing endothelial nitric oxide availability and microvascular tone, consequently affecting follicular and luteal perfusion [138]. These pathways collaborate to link vesicle biogenesis with vesicle function. Temporary bleeding may adversely affect oocyte competence, embryo quality, and implantation, while also inducing persistent redox disease across cycles.

### 5.2. Cytokine Amplification, Immune System Reprogramming, and Inflammation

Chronic sterile inflammation is the primary indicator of endometriosis, and extracellular vesicles facilitate communication about this inflammation. Lesion-derived extracellular vesicles are replete with pro-inflammatory cytokines, receptors, and signalling microRNAs that induce immune cells to adopt a more tolerant phenotype and facilitate lesion support [139]. MiR-21, miR-146a, and miR-155 are the most extensively researched microRNAs. They alter the macrophage phenotype from M1 to M2 by inhibiting SOCS1, IRF5, and SHIP1. M2 macrophages facilitate angiogenesis and fibrosis by releasing their own extracellular vesicles enriched with VEGF, TGF-β, and IL-10. To build a resilient set-point for cytokine production that endures negative feedback, extracellular vesicles carry HMGB1, S100A8/A9, and galectins, which aggregate pattern-recognition receptors and amplify NF-κB signalling [140]. EV cargo reduces effector T-cell priming and promotes Treg proliferation in dendritic cells by modifying antigen presentation to favour tolerogenic profiles (low CD80/CD86, high PD-L1).

NK cells and T lymphocytes are also influenced by extracellular vesicle-mediated immunological suppression. Exosomal PD-L1, Galectin-9, and FasL derived from endometriotic stromal cells bind to inhibitory receptors on cytotoxic lymphocytes, therefore diminishing the secretion of IFN-γ and the apoptosis of immune effector cells [141]. Similarly, vesicular miR-214 and miR-23a inhibit perforin production and NK-cell degranulation. The final outcome is a microenvironment that is immunologically conducive and capable of sustaining the life of ectopic tissue. Concurrently, EV-mediated interactions among fibroblasts, mesothelial cells, and macrophages lead to the exacerbation of inflammation [142]. PF-EVs rich in S100A8/A9 and HMGB1 prompt mesothelial cells to release IL-6, IL-8, and MCP-1, therefore recruiting and activating macrophages via TLR4/NF-κB signalling pathways. In vitro studies demonstrate that GW4869, an inhibitor of neutral sphingomyelinase, markedly decreases cytokine levels in coculture systems when vesicle release is inhibited [143]. This underscores the pivotal function of EVs in maintaining the inflammatory circuit. Furthermore, EV-related complement components (C3, C5 cleavage fragments) and tissue factor initiate coagulation-inflammation interactions (thrombin–PAR signalling), increasing vascular permeability and leukocyte extravasation while forming a fibrin scaffold that stabilises initial wounds.

### 5.3. Pain Sensitisation, Neurogenesis, and Angiogenesis

Active neovascularisation and nerve infiltration are essential for the maintenance and manifestation of endometriosis lesions. EVs are increasingly recognised as crucial for angiogenesis and neurogenesis. The clusters of VEGF-A, ANGPT1, FGF2, and miR-126/miR-132 found in vesicles from endometriotic epithelial and stromal cells stimulate VEGFR2/eNOS/PI3K–AKT signalling in endothelial cells, facilitating migration and tube formation. Parallel proteomic studies indicate a higher prevalence of integrins αvβ3, CD105, and annexins [7]. These chemicals facilitate the adhesion of endothelial cells and alter the configuration of the cytoskeleton. Vesicular MMPs modify the basement membrane to facilitate vessel ingrowth. The lipid cargo of extracellular vesicles, including S1P, LPA, and oxidised phospholipids, further activates S1P and LPA receptors to enhance endothelial permeability and sprouting [17]. Metabolic changes caused by EVs in endothelial cells (upregulation of glycolysis via PFKFB3) promote angiogenesis by securing ATP supply for migration and lumen formation in hypoxic environments.

Vesicular NGF, BDNF, and neuregulin-1 together promote neurogenic signalling in conjunction with miRNAs that target the semaphorin and neuropilin families, which govern axon guidance. Upon the uptake of these vesicles by sensory neurones, the levels of TRPV1 and Nav1.8 increase [96]. This enhances the sensitivity of pain pathways and aids in identifying the molecular aetiology of dysmenorrhea and persistent pelvic pain. Peritoneal macrophage-derived extracellular vesicles unexpectedly facilitate this process by releasing vesicles abundant in IL-6 and miR-21. These chemicals stimulate endothelial cells and fibroblasts to produce STAT3-dependent neurotrophic factors, hence reinforcing the neuroangiogenic feedback loop [144]. Concurrently, EVs create a neuroimmune niche that sustains pain even after lesion debulking by modifying Schwann cell morphologies and facilitating perineural invasion. The treatment proposes obstructing the EV route, using nSMase2 inhibitors or anti-integrin/tetraspanin methods, as a supplementary approach to surgery or hormone therapy to simultaneously sever both blood and nerve supply. This EV-driven vascular-neural connection clarifies the inadequacy of analgesic responses and the relationship between lesion vascular density, nerve fibre scores, and pain intensity.

ECM Remodelling, EMT, and Fibrosis EVs are crucial for the fibrotic remodelling of both ectopic and eutopic tissues, which is essential for the advancement of endometriosis. miR-21-5p, miR-29c, miR-31-5p, and miR-214 are extracellular vesicle-associated microRNAs that facilitate fibrotic transformation by regulating the PI3K/AKT, YAP/TAZ, and TGF-β/SMAD signalling pathways [139]. These vesicles induce recipient stromal fibroblasts to excessively produce FN1, α-SMA, and COL1A1, resulting in extracellular matrix deposition and increased tissue rigidity. Lesion-derived extracellular vesicles also contain fibronectin, LOX, and latent TGF-β. These chemicals mechanically incorporate into the ECM to enhance fibrotic stability [145]. Crucially, EV-integrin codes (e.g., αvβ3, α5β1) confer organotropism and direct vesicles to fibroblast-dense niches; subsequent internalisation fosters RhoA/ROCK-dependent contractility and FAK/Src activation, thereby augmenting tensional homeostasis and securing a profibrotic transcriptional state via nuclear programmes MRTF-A and TEAD [146].

EVs alter the ratio of MMPs to TIMPs, hence directly influencing the matrix’s processes of synthesis and degradation. In endometriosis-derived extracellular vesicles, TIMP expression is simultaneously suppressed while MMP-2 and MMP-9 remain active, promoting invasion across the ovarian and peritoneal surfaces. These matrix-modifying vesicles induce the EMT via transporting Snail, Twist, and Zeb1 mRNAs, as well as miR-200 family inhibitors. This results in epithelial cells losing E-cadherin and acquiring vimentin [147]. This EMT-like alteration enhances the mobility and invasiveness of lesions, facilitating their growth and penetration into the tissue, particularly in ovarian endometriomas and rectovaginal nodules. Fibrogenic extracellular vesicle activity occurs in the eutopic endometrium, where repeated hypoxic and inflammatory stress prompts the vesicular production of miR-29 family suppressors, therefore affecting collagen homeostasis and leading to progesterone resistance and uterine stiffness. These processes produce fibrotic matrices characterised by a paucity of blood vessels and an abundance of adhesions [131]. These beds exhibit resistance to pharmacological treatment and surgical intervention, increase the likelihood of pain recurrence, and impede the uterine ability to alter its shape, so hindering implantation. This indicates that EVs are essential upstream targets to avert the progression of mild inflammatory lesions into hard fibrotic illness.

### 5.4. Hormonal Interactions, Epigenetic Variation, and Resistance to Progesterone

Endometriosis is defined by progesterone resistance and oestrogen dominance. Recent investigations demonstrate that EV-mediated signalling is crucial in regulating these hormonal abnormalities. Extracellular vesicles from lesions and the endometrium include miRNAs that target co-regulators of steroid hormone receptors, such as NCOA1, SRC-1, and HSD17B2 [148]. The miRNAs include miR-29c, miR-135a, miR-200b, and miR-221. These alterations maintain the tissue in a hyperestrogenic, proliferative condition by inhibiting the function of the PR-B while preserving the activity of the ER-β [149]. Moreover, EV cargo alters aromatase pathways and 11β-HSD enzymes, hence increasing local oestrogen production and progesterone deactivation. This delineates the eutopic endometrium from luteal signals that typically initiate the process of decidual competence.

Epigenetic modifiers are used by extracellular vesicles to alter chromatin structure. Vesicle cargo, including DNMT1, HDAC1, and TET2 fragments, together with miR-29 family inhibitors, facilitates global DNA methylation alterations that enhance estrogen-responsive gene expression and suppress PR target genes [150]. This epigenetic drift disseminates by EV-mediated transfer, resulting in hormone resistance occurring simultaneously at both eutopic and ectopic locations. Furthermore, the exposure of endometrial cells to these vesicles modifies the expression of histone acetylation markers (H3K27ac, H4K16ac), thereby establishing a connection between transcriptional reprogramming and vesicular acetate metabolism via ACSS2 [151]. Anti-receptive extracellular vesicle miRNAs (such as miR-145-5p) convert hormonal resistance into functional implantation failure at the embryo–endometrium interface by inhibiting the NOTCH and LIF–STAT3 pathways in the trophectoderm and luminal epithelium. These results indicate that extracellular vesicles function as the critical mechanistic connection between the biology of endocrine receptors and clinical outcomes, suggesting that restoring receptivity in endometriosis may require modifications in vesicle cargo rather than simple hormonal changes.

## 6. EV-Derived Biomarkers in the Context of IVF: From Implantation Dynamics to Oocyte Competence

Endometriosis impairs reproductive potential at every critical phase of assisted reproduction, from folliculogenesis to embryo implantation. EVs have become preferred real-time markers of these processes owing to their stability, specificity, and capacity to contain the molecular signature of the reproductive tract [152]. They associate molecular pathology with measurable IVF outcomes, such as oocyte maturity, fertilisation rate, embryo morphokinetics, implantation, and live birth, by clarifying the mechanisms of the ovarian follicle, endometrium, and embryo. This section clarifies the relationship between the molecular structure of EVs and cellular dysfunctions, along with clinical effects, including current research that substantiates their role as multifunctional biomarkers in infertility linked to endometriosis [153]. Besides passive association, EVs generate signals that may be acted upon, enabling iterative decision-making due to their fast changes in response to treatment, stimulation, and cycle phase. When examined within a standardised pre-analytic framework (MISEV-compliant isolation, platelet depletion, exogenous spike-ins, endogenous reference miRNAs), EV readouts can be tracked over time to predict an adverse response, such as impending luteal phase insufficiency or oxidative spikes during stimulation [154]. This implies that EVs can recognise at-risk people and choose the optimal period to provide immunomodulators, antioxidants, or elect to defer transmission. The subsequent subsections provide a comprehensive examination of the correlation between each IVF checkpoint and EV biology, as well as the incorporation of vesicular signals into standardised clinical algorithms pertinent to both individual patient management and institutional protocols.

### 6.1. The Competence of Oocytes and the Follicular Microenvironment

The ovarian follicle consists of granulosa, cumulus, and theca cells that operate together as a meticulously organised ecosystem. They send and receive signals via paracrine and juxtacrine pathways, many of which are mediated by EVs. Extracellular vesicles in follicular fluid modulate oocyte maturation under normal physiological conditions by transporting growth factors (GDF9, BMP15, EGF-like ligands), miRNAs (miR-132, miR-320a, and miR-214), and proteins linked to steroidogenesis. This regulatory network is impaired in women with endometriosis [155]. Follicular extracellular vesicles include an abundance of metabolic enzymes (LDHA, ACSS2, ENO1) and microRNAs associated with oxidative stress (miR-23a, miR-21-5p), impairing ATP synthesis, mitochondrial functionality, and cumulus-oocyte interaction. Co-culture studies indicate that EVs derived from endometriosis follicles induce apoptosis, mitochondrial fragmentation, and reduced oestradiol synthesis in healthy granulosa cells, hence impeding the cytoplasmic and nuclear maturation of oocytes [156]. Single-follicle analyses corroborate these findings by revealing correlations between extracellular vesicle miRNA levels (e.g., elevated miR-21-5p/miR-23a) and mechanistic irregularities indicative of diminished MII rates and increased aneuploidy risk, encompassing reduced mitochondrial membrane potential in oocytes, disrupted spindle assembly, and impaired metaphase plate alignment. The vesicular shift diminishes HAS2/PTX3 expression, hence hindering cumulus development, leading to insufficient cumulus matrix synthesis and disrupted oocyte-somatic signalling during final maturation [157].

Furthermore, in follicular extracellular vesicles, vesicular proteins like S100A8/A9, Annexin A2, and Peroxiredoxin 3 serve as oxidative stress amplifiers and redox regulators in chronic inflammatory conditions. These compounds activate the NF-κB and p38 MAPK pathways in granulosa cells via their engagement with RAGE and TLR4. This alters the transcriptome to augment pro-inflammatory responses and diminish the probability of cell death [158]. These settings, ironically, boost survival while hindering development potential. Consequently, the yield of metaphase-II oocytes diminishes, the fertilisation rate declines, and the prevalence of aberrant morphokinetic patterns during the first phases of embryonic development escalates. Furthermore, endometriosis is linked to reduced levels of follicular EV-associated miR-320a, a microRNA generally involved with efficient communication between cumulus and oocyte [159]. This pertains to problems of cumulus development and decreased clinical pregnancy rates. These findings provide clinical support for pre-retrieval optimisation (e.g., targeted antioxidants, omega-3 fatty acids, CoQ10, or metformin in insulin-resistant phenotypes) alongside EV monitoring: a reduction in the miR-21-5p/miR-23a ratio and the restoration of miR-320a in follicular EVs may serve as preliminary indicators of enhanced oocyte competence, aiding in the decision to either proceed with a fresh transfer or adopt a freeze-all strategy during optimisation [160].

### 6.2. Embryo Morphokinetics and Extracellular Vesicles: Modifying Developmental Timing via Molecular Interference

Embryo morphokinetics involves transcriptional accuracy and molecular energy dynamics. It includes the phases of cleavage, compaction, and blastocyst formation. EVs directly impact early embryonic development by their molecular content and indirectly by modifying the uterine and follicular environments that regulate this process. UF-EVs are essential during the preimplantation phase as they aid in the blastocyst’s readiness for implantation [161]. EVs produced from embryos engage in a feedback mechanism that synchronises maternal readiness with embryonic development by modulating cytokine concentrations and endometrial adhesion molecules.

UF-EVs transport miRNAs, including miR-30d, miR-200b, and miR-148b, from the receptive endometrium to the embryo to promote its growth. These miRNAs govern trophectoderm adhesion, metabolism, and epigenetic programming. These vesicles include lipid mediators, including as prostaglandins and phosphatidylserine derivatives, which promote embryo hatching and zona pellucida softening [162]. In endometriosis, UF-EVs demonstrate a notable modification in their cargo composition. The downregulation of miR-30d reduces trophoblast adhesion, while the elevation of miR-145-5p, miR-155-5p, and miR-199a-3p impedes the activities of NOTCH1/2, VEGFA, and E-cadherin. Time-lapse imaging studies reveal that embryos exposed to these vesicles in vitro have irregular compaction patterns, asynchronous divisions, and protracted cleavage rates [31]. Subsequent research associates UF-EV inflammatory indicators (elevated S100 proteins, HSP70) with modified ICM/TE distribution and increased trophectoderm caspase-3 activation, resulting in diminished blastulation rates and impaired inner cell mass quality. Morphokinetic models misclassify some embryos as viable, especially in the presence of EV-mediated anti-implantation signals. The UF-EV profile may improve selection, enabling cryopreservation and cycle deferral, instead of performing a low-probability transfer, by detecting inconsistencies between visually “normal” development and negative uterine signals [163].

In addition to miRNA effect, alterations in extracellular vesicle protein cargo, namely deficits in annexins, galectins, and integrins, affect blastocyst polarity and metabolic adaptation. Electric cars displaying an inflammatory profile are defined by the presence of S100 proteins and HSP70. They may activate stress pathways in trophectoderm cells, leading to cell death and impeding ICM/TE growth. The molecular miscommunication of extracellular vesicles causes measurable morphokinetic changes that link minor variations in cargo to substantial differences in in vitro fertilisation results. This organisation fervently promotes the integration of EV profiling with embryo morphokinetic scoring, therefore offering a molecular rationale for the visual patterns seen during in vitro development. Clinics may use a dual-signal protocol: initiate a new transfer only if both the UF-EV receptivity index and morphokinetics are favourable. Otherwise, adopt a freeze-all approach. Integrating these signals into predictive models, such as gradient-boosted trees using maternal factors, embryo timings, and EV miRNA intensities, should improve implantation prediction beyond the efficacy of each method alone.

### 6.3. Endometrial Receptivity and Implantation: Extracellular Vesicles as Molecular Indicators of Efficacy

The implantation period may be the most vulnerable to extracellular vesicles in reproduction. EVs are acknowledged as the principal agents enabling communication between maternal and embryonic tissues, therefore priming the endometrium for receptivity. During the mid-secretory phase, the receptive endometrium releases extracellular vesicles distinguished by a specific miRNA profile. This miRNA signature, mostly including miR-30d, miR-200a/b, miR-141, and let-7 family members, modulates the expression of trophoblast genes to enhance adhesion and invasion [70]. These vesicles include proteins like as integrins (αvβ3), tetraspanins, and SPP1 that directly interact with embryonic surface receptors, promoting adhesion and stability. Extracellular vesicle lipids promote the alteration of the embryonic membrane, augmenting the density and robustness of microvilli, while metabolic effectors, including glycolytic enzymes and amino acid transporters that optimise embryonic bioenergetics, are conveyed to the hypoxia-graded endometrial environment through vesicle fusion or internalisation [117].

This synchronised EV-mediated process deteriorates in endometriosis. Elevated concentrations of miR-145-5p, miR-155-5p, and miR-451a in extracellular vesicles derived from uterine fluid inhibit the NOTCH, STAT3, and LIF signalling pathways, which are essential for receptivity and decidualisation. The increased levels of vesicular proteins associated with immunological regulation, such as complement components, S100A9, and CD142 (tissue factor), signify a shift towards a pro-coagulant, pro-inflammatory environment that obstructs successful implantation [31]. Clinical studies demonstrate that UF-EV miRNA panels surpass conventional transcriptomic assays in differentiating between implantative and non-implantative endometria, with AUC values above 0.9. This suggests that EVs not only represent the window of implantation but also define it mechanically. Decisions guided by UF-EV may shorten the time to conception, avoid unnecessary transfers, and mitigate the financial and emotional burdens of repeated failed cycles [116]. Clinicians may ascertain a patient’s specific WOI and efficiently synchronise transfer by conducting serial collection of UF-EVs throughout simulated luteal phases. Alternatively, they may identify a temporally refractory endometrium and implement corrective measures (such as anti-inflammatory treatments, modified progesterone exposure, and GnRH modulation), using EVs to verify physiological reversal prior to transfer.

### 6.4. Menstrual-Blood Extracellular Vesicles and Serum as Indicators of Systemic Fertility

Menstrual blood and serum EVs provide a thorough insight for assessing disease activity and reproductive readiness. MicroRNAs produced from serum extracellular vesicles, such as let-7b-3p, miR-22-3p, and miR-320a, are substantially correlated with the results of in vitro fertilisation pregnancies. These miRNAs represent the cumulative impacts of hormonal signalling, oxidative stress, and inflammation [122]. The reliability of increased serum EV miR-22-3p and miR-320a levels as non-invasive prognostic indicators is shown by their capacity to forecast clinical pregnancy and live birth, with AUC values above 0.8. These indicators assess the effectiveness of the treatment. A decrease in pro-inflammatory EV miRNA levels after lesion excision or treatment signifies clinical improvement and improved ovarian response, acting as a molecular marker for the commencement of ART [51]. Serum EV panels may influence stimulation intensity and set expectations during pre-IVF consultations. When used with standard laboratory evaluations like as AMH, AFC, TSH, and vitamin D, they provide composite prognostic indices that surpass the efficacy of any singular indicator [164].

MB-EVs provide a periodic molecular biopsy of the endometrium, reinforcing this systemic viewpoint. Their miRNA content, namely miR-21-5p, miR-23a, miR-29c, and miR-4443, signifies the regeneration potential of the endometrium by indicating active fibrotic and proliferative processes [165]. Before IVF, longitudinal MB-EV profiling may provide real-time assessment of endometrial viability, hence enhancing luteal support and stimulation protocols. Improved implantation rates are significantly associated with the normalisation of MB-EV signatures (adjusted miR-30d/miR-200 ratio, reduced oxidative markers) throughout medical therapy, highlighting EVs as molecular indicators of treatment efficacy [166]. The MB collection may be performed at home and is scalable. Standardised kits (low-protein adsorptive plastics, stabiliser tubes) may diminish noise before analysis, while cloud-based reporting of EV indices offers physicians remote access to decide whether to continue preparing for medical treatment, transition to freeze-all, or proceed with fresh transfer [167]. This converts menstrual cycles into significant milestones instead of mere waiting periods, so matching ART timing more accurately with genuine molecular readiness.

### 6.5. Integrating EV Biomarkers into the Decision-Making Process for Art

The therapeutic significance of EVs in IVF arises from their capacity to amalgamate diagnosis, prognosis, and personalisation. Clinicians may create a detailed fertility profile by examining vesicular signals in several matrices, such as serum, follicular fluid, menstrual blood, and uterine fluid [168]. Follicular EVs may signify oocyte quality, uterine fluid EVs may ascertain the ideal time for embryo transfer, and serum EV miRNA patterns may disclose the existence of inflammatory or metabolic endometriosis in a patient. This integrated approach allows EV-based clinical algorithms to facilitate personalised treatments, including antioxidant support, anti-inflammatory pre-treatment, tailored stimulation, or freeze-all cycles depending on the endometrium’s preparedness [47]. A three-gate framework may be used in practical applications. Gate 1 (Baseline Serum EV Index) establishes the pre-treatment and protocol intensity. Gate 2 (Follicular EV Fitness) evaluates the authorisation of the trigger or the provision of extended maturation support. Gate 3 (UF-EV Receptivity) assesses the approval for fresh transfer or the deferral for cryo-transfer. Validated panels, with decision curves that illustrate a net clinical benefit relative to traditional therapy, are used to define the criteria for each gate [169].

Moreover, EVs provide benefits for ongoing monitoring throughout the IVF process owing to their availability and reliability. A minimally intrusive sample of pre-transfer UF-EVs may evaluate receptivity, mid-cycle follicular EVs might assess oocyte growth, and a baseline serum-EV analysis could monitor disease activity. The integration of machine-learning algorithms that assess EV molecular patterns with hormonal and imaging data to provide predictive ratings for embryo implantation or live birth might revolutionise precision medicine in IVF. To avoid overfitting, these models need calibration and external validation, inclusion of factors associated with menstrual phases and hormone intake, and compliance with TRIPOD+MISEV reporting standards. With these precautions, EV analytics may evolve from experimental supplements to standard, sponsored evaluations included into EHRs, offering clinicians clear, understandable, and verifiable recommendations.

## 7. Diagnostic and Prognostic Potential of EV-Based Biomarkers in Endometriosis-Associated Infertility

The therapeutic significance of EV biology relies on the accuracy of its evaluation for prognostic and diagnostic purposes. In the last decade, much research has shown that molecular fingerprints from extracellular vesicles, including miRNAs, proteins, and lipid mediators, may surpass various commonly used biomarkers in IVF and endometriosis. EVs contain disease-specific cargo that is protected from enzymatic destruction [170]. This makes them resilient and easily identifiable in biofluids, unlike soluble cytokines or hormones, which are transient and significantly distinct. Their lipid bilayer preserves the stoichiometry of miRNA–protein complexes, and their origin from lesions, endometrial, and immune cells ensures biological specificity [171]. This section summarises the current information about the diagnostic and prognostic uses of EVs, emphasising translational readiness, matrix selection, and analytical robustness. The mechanistic coherence provided by EV assays beyond mere signal detection, the same vesicular miRNAs and proteins that define disease are also located upstream of pathogenic pathways (e.g., NF-κB, PI3K/AKT, and TGF-β/SMAD), offering a face validity lacking in traditional markers [172]. Moreover, EVs provide intrinsic benefits for longitudinal monitoring, enabling the observation of temporal variations (improving, staying stable, or declining) rather than only evaluating a single instant in time. The temporal component is essential in ART due to the frequency of treatment adjustments and inter-cycle fluctuations. Ultimately, since EVs may be extracted from several matrices, including serum/plasma, menstrual blood, follicular fluid, and uterine fluid, clinicians can develop multi-compartment panels that include local receptivity and systemic inflammation [173]. This integrates disparate diagnoses into a unified, actionable summary that guides therapy, timing, and transfer strategy. Table 2 shows the diagnostic and prognostic accuracy (AUC, sensitivity, specificity) of EV-based panels for finding endometriosis and predicting ART outcomes. This gives a complete picture of how well these panels work across different matrices and endpoints.

An overview of diagnostic and prognostic models for endometriosis and ART based on the payload of EVs. Examples of measures include sensitivity, specificity, and area under the curve. Most panels include two to five proteins or miRNAs derived from EVs, present in menstrual blood, serum, plasma, or uterine fluid. The most optimal predictive performances (AUC > 0.84) were seen in EVs derived from serum and uterine fluid. UF denotes uterine fluid, MB signifies menstrual blood, ROC refers to receiver-operating characteristic, and CI represents confidence interval.

According to Table 2, the diagnostic accuracy of EV-based assays for endometriosis is now as good as or better than that of many standard clinical and imaging tests. Serum and plasma EV-derived miRNAs, particularly those identified by Muraoka et al. (2024) and Sadati et al. (2024), achieved AUC values exceeding 0.80 for distinguishing fertile from infertile women or predicting pregnancy outcomes [51,72]. Because they were so close to the implantation microenvironment, uterine-fluid panels, especially the ones from Apostolov et al. (2025), had almost perfect discriminative power (AUC ≈ 0.9) [32]. Conversely, menstrual-blood EV profiles provide a non-invasive alternative for early disease detection, exhibiting high reproducibility and sensitivity (Ji et al., 2024; Gurung et al., 2025) [35,174]. These findings collectively validate EV cargo as a dependable diagnostic substrate and demonstrate significant potential for pre-ART prognostication, notwithstanding methodological variability.

### 7.1. Diagnostic Utility: Differentiating Endometriosis from Infertility Related to Endometriosis

A plethora of high-quality studies indicates that circulating and local EVs possess distinct molecular signatures that may differentiate women with endometriosis from healthy controls or those with unexplained infertility. Panels including miR-22-3p, miR-320a, members of the miR-200 family, miR-21-5p, and miR-451a have consistently shown diagnostic accuracy in plasma and serum, with area under the ROC curve (AUC) values between 0.78 and 0.94 [139]. These miRNAs regulate oxidative stress, fibrosis, and immune activation pathways critical to the pathophysiology of endometriosis. Serum EV miR-22-3p and miR-320a concentrations are decreased in infertile individuals with little or no pathology, but they are raised in cases of advanced-stage endometriosis [74]. This dynamic range exceeds that of traditional markers like CA-125 or IL-6, which show considerable background noise and are inadequate in differentiating between distinct phases. A dual-level EV screening (a comprehensive serum EV panel followed by a confirmatory uterine-fluid or menstrual-blood EV assay) can minimise unnecessary imaging and invasive diagnostics in clinical triage by ensuring high sensitivity for early disease detection while preserving specificity for clinically significant lesions [175]. Moreover, using cargo that assesses fibrosis, neuroangiogenesis, and coagulation, EVs may differentiate between phenotypes (e.g., ovarian endometriomas vs. deep infiltrating disease) and provide etiological insights unattainable by traditional biomarkers.

The varying amounts of miR-21-5p, miR-29c, miR-4443, and miR-23a in MB-EVs provide diagnostic accuracies of 90% for endometriosis identification. These molecules, prominent in the PI3K/AKT and TGF-β/SMAD pathways, signify active fibrogenic and metabolic remodelling, as well as the presence of disease. MB-EV analysis is an entirely non-invasive diagnostic technique that does not need blood extraction or laparoscopy [176]. Proteomic research enhances specificity by revealing that lesion-derived EVs exhibit S100A8/A9, Annexin A2, and Peroxiredoxin 2, which are either missing or expressed at minimum levels in controls. The integration of EV-protein and EV-miRNA to develop multi-omic classifiers might get a specificity of over 95% [158]. Telemedicine workflows may include at-home sample collections using standardised kits (stabilisers, pre-labelled tubes, QR-coded chain-of-custody) to provide population-scale screening that is broadly accepted by patients. Non-invasive MB-EV testing may detect high-risk adolescents and young adults for prompt referral, reducing significant diagnostic delays in this demographic [177]. This may inhibit the development of severe disease characteristics that might hinder future pregnancy. A continuous pattern over cycles, rather than an isolated high value, may enhance diagnostic confidence and substantiate clinically significant disease, since MB-EV cargo fluctuates with cycle phase and hormonal exposure.

From a translational perspective, EV diagnostics might revolutionise patient triage. Women with dysmenorrhea and subfertility may undergo serum or menstrual extracellular vesicle testing prior to imaging or laparoscopy. A negative test may rule out disease and save unnecessary operations, but a positive EV signature would need early contact with reproductive specialists and enable customised ART planning [178]. This non-invasive, progressive approach corresponds with the clinical shift towards ‘biopsy-free’ diagnosis of endometriosis, markedly decreasing the diagnostic duration from years to months. In areas lacking easy access to laparoscopy, EV testing may function as a preliminary evaluation to ascertain case urgency and prioritise surgical resources for the most essential circumstances. This improves the fairness of treatment [179]. Ultimately, EV-driven pathways exhibit a decrease in unnecessary therapies and a more rapid shift to successful therapy. This supports value-based methodologies advantageous for patients, clinicians, and healthcare systems, since payment structures increasingly need outcomes-oriented rationale.

### 7.2. Prognostic Utility: Predicting Reproductive Outcomes and ART Efficacy

In addition to diagnosis, EVs include predictive information that may affect IVF results. Studies have shown a significant correlation among ovarian response, embryo quality, implantation, and live birth, alongside the detection of EV miRNA panels in blood and uterine fluid [180]. Serum concentrations of EV miR-22-3p, miR-320a, and let-7b-3p before to ovarian stimulation may predict the number of mature oocytes retrieved and subsequent pregnancy results. The AUCs for these values range from 0.80 to 0.88. Patients with consistently low levels of EV miR-320a, regardless of age or AMH, often demonstrate worse oocyte quality and diminished blastulation rates [181]. Follicle-fluid extracellular vesicle miR-21-5p and miR-23a exhibit an inverse correlation with embryo cleavage synchrony and metaphase-II yield, suggesting that extracellular vesicle-based follicular evaluations are superior indicators of oocyte health compared to static hormone levels. These indicators may be evaluated throughout cycles to see whether pre-IVF optimisation (anti-inflammatory therapy, antioxidants, and lifestyle adjustments) is producing a biological effect. They provide unbiased criteria for deciding whether to proceed with stimulation or to persist with preparatory actions [182].

Uterine-fluid EVs have emerged as important indicators of receptivity during implantation. Distinct miRNA signatures, particularly downregulated miR-145-5p, upregulated miR-30d, and miR-200b, distinguish responsive from non-receptive endometria in women undergoing FET, achieving an AUC of 0.9 and surpassing ERA-based transcriptome assessments [52]. These vesicular markers display functional activity and indicate transcriptional readiness. Receptive-phase UF-EVs promote adhesion and outgrowth in vitro, but non-receptive EVs impede these processes. Consequently, EV profiling might replace invasive endometrial biopsies by offering a non-invasive, same-cycle method to assess implantation likelihood. Given the length of progesterone presence, EV signals remain interpretable even throughout the injection of exogenous hormones [71]. This suggests that readiness evaluations may occur before the initiation of IVF procedures. UF-EV indices provide dual-signal decision points when used with time-lapse morphokinetics. This improves the overall live birth rate and minimises unnecessary fresh transfers by maximising the synchronisation of the transfer with an endometrium that is sufficiently prepared for acceptance [183].

The predictive stability of EVs remains constant across treatment cycles. Longitudinal monitoring indicates that improved ovarian and implantation parameters are associated with the normalisation of extracellular vesicle profiles after surgery or GnRH agonist therapy (e.g., restoration of miR-320a and reduction in pro-inflammatory miR-21-5p) [184]. Consequently, EVs serve as dynamic feedback systems and indications of therapy success. Clinicians may assess a patient’s readiness for IVF or the need for further medical intervention prior to stimulation by temporal monitoring. The fast reemergence of negative EV signals after surgery may suggest a quick recurrence, requiring vigilant monitoring or further medical action. Conversely, a prolonged elevation in ER molecular profile may enable the commencement of ART at an earlier stage, hence reducing the interval prior to sickness recurrence. When used together, these applications convert EVs from passive predictors into active navigators of ART duration and intensity [185].

### 7.3. Reproducibility and Analytical Resilience

For EV-based biomarkers to be dependable, the used techniques must demonstrate significant consistency. Unlike free-circulating nucleic acids, extracellular vesicles need careful consideration of pre-analytical factors: yield and purity are affected by sample volume, centrifugation parameters, control of haemolysis, and freeze–thaw cycles [186]. The MISEV 2023 principles have enabled substantial progress via the establishment of alignment. Differential ultracentrifugation, size-exclusion chromatography, and immunoaffinity isolation facilitate the production of reproducible particle populations enriched in CD9, CD63, CD81, and TSG101, free from endoplasmic reticulum contaminants such as calnexin [187]. Microfluidic devices that inhibit precipitation can separate extracellular vesicles from microlitre samples in 15 min, making them beneficial for same-day diagnostics and enabling clinical scalability. To improve standardisation, labs have to use orthogonal characterisation (NTA + bead-based flow + EM), record particle-to-protein ratios, and oversee platelet depletion processes. These procedures reduce noise before analysis and enable the comparison of results among labs [188].

Normalisation methods are crucial for analytical consistency. Exogenous spike-ins (cel-miR-39) and endogenous reference miRNAs (e.g., miR-16-5p, miR-191-5p) provide comparability across samples from diverse populations. In distinct cohorts, machine-learning methodologies using EV miRNA panels, such as LASSO logistic regression, elastic net, or random forest classifiers, have shown accuracies over 90% [189]. Calibration plots and decision-curve analysis translate model outputs into clinically relevant probabilities, whilst layered cross-validation including menstrual-phase and hormone-use variables mitigates overfitting. Furthermore, multi-matrix models including signals from menstrual blood and serum extracellular vesicles provide enhanced diagnostic capabilities, with the former representing localised endometrial remodelling and the latter signifying systemic inflammation [190]. Obtaining regulatory approval necessitates the use of pre-registered analytical protocols and external quality assurance mechanisms, including inter-laboratory ring trials using standardised reference materials. These analytical pipelines provide extracellular vesicles as reliable, repeatable molecular tests that meet certification standards (such as ISO 15189) and are ready for incorporation into clinical diagnostic protocols.

EV-based assays consistently exhibit enhanced sensitivity, specificity, and stability relative to established markers such as CA-125, IL-6, TNF-α, and VEGF. Extracellular vesicle microRNAs maintain disease-specific profiles independent of hormonal fluctuations, while CA-125 levels fluctuate with the menstrual cycle and are elevated in benign conditions such as fibroids and adenomyosis [191]. Moreover, EVs demonstrate enhanced efficiency relative to cell-free microRNAs, which deteriorate quickly and exert systemic rather than localised biological effects. Unlike transcriptomic or proteomic endometrial evaluations such as ERA and ReceptivaDx, EV tests are more efficient, may be conducted many times during the same cycle, and do not need tissue sampling [192]. Moreover, EVs are more congruent with dynamic processes like as receptivity and implantation, since they encapsulate intercellular communication rather than cellular expression. Due to this functional focus, EV markers often exhibit a more robust connection with outcomes (such as implantation or live birth) than composite tissue assessments.

Innovative multi-omic approaches are merging EV miRNAs, proteins, and metabolites to provide accurate phenotyping. An integrative analysis of serum extracellular vesicle miRNAs and lipids (sphingomyelins, phosphatidylserine species) enhances the classification of early-stage disease by including both transcriptional and metabolic dimensions of pathology [193]. Spectral-mass spectrometry of EV phosphoproteins has shown that MAPK and AKT signalling pathways are activated in a disease-specific manner. This may be used to classify people according to their propensity to react to certain medications, such as PI3K inhibitors or GnRH analogues. Pilot studies suggest that fusion models (omics, ultrasound features, and clinical history) substantially improve AUC and decrease false positives compared to any individual modality. As costs diminish and sample-to-answer platforms advance, multi-omic EV assays may replace obsolete indications that lack specificity and adaptability in clinical environments, functioning as key tests to aid in ART planning and surgical decision-making.

### 7.4. Clinical Translation and Prognostic Scoring Systems

Validated EV-based grading systems are essential for the transition from discovery to routine use. Numerous organisations have proposed logistic regression or neural network methods to develop composite indices for forecasting IVF success, including EV miRNA levels, clinical features, and hormonal variables. The Endometrial EVRS utilises the ratios of miR-30d to miR-145 to ascertain appropriate implantation times [194]. The Serum EV Fertility Index utilises EV miR-320a, miR-22-3p, and BMI to predict clinical pregnancy with an accuracy of over 85%. These indices function as versatile tools for patient classification, since they may be reconfigured for different populations. In clinical deployment, risk bands (green, amber, and red) may prompt standard measures such as initiating a new transfer, pausing all operations for optimisation, or delaying for medical intervention. Decision-curve analysis facilitates the modification of parameters to optimise net benefit (true positives minus weighted false positives). Integrating these evaluations into electronic health records with clear outputs that specify the key elements affecting the recommendation bolsters professional confidence and patient understanding [195].

Regulatory harmonisation and standardised reference ranges are crucial for the effective translation of therapeutic practices in the future. Implementation studies must include cost-effectiveness, integration with electronic health records, and the repeatability of analytical results [196]. EV assessments must be integrated into standard ART protocols, which include uterine fluid analysis before transfer for receptivity confirmation, follicular sampling during retrieval for oocyte competence evaluation, and blood or menstrual collection on cycle day 2 for baseline assessment. This tiered EV-based methodology might transform the IVF process by replacing imprecise timing evaluations with milestones defined by molecular accuracy. This would improve success rates while decreasing the number of cycles and psychological stress. Health-economic models indicate that even little improvements in the per-cycle live birth rate and a decrease in unnecessary transfers provide cost savings and accelerate the time to conception, hence supporting the case for reimbursement and wider use. Essential stages in the translational roadmap will include executing prospective, multicentre validation studies with explicit targets (live birth, sustained implantation) and partnering with diagnostic companies to provide kit-based, CLIA-compliant assays.

### 7.5. Models for Clinical Translation and Prognostic Assessment

Validated EV-based grading systems are crucial for the shift from discovery to everyday practice. Various organisations have developed logistic regression or neural network models to create composite indices for predicting IVF success, including EV miRNA levels, clinical characteristics, and hormonal factors. The EVRS employs the ratios of miR-30d to miR-145 to determine optimal implantation timings [197]. The SEFI employs EV miR-320a, miR-22-3p, and BMI to forecast clinical pregnancy with over 85% accuracy. These indices serve as adaptable instruments for categorising patients, since they may be recalibrated for other populations. In clinic deployment, risk bands (green, amber, and red) may initiate typical actions such as proceeding with a fresh transfer, halting all activities for optimisation, or deferring for medical suppression [198]. Decision-curve analysis allows for the adjustment of thresholds to maximise net benefit, defined as true positives minus weighted false positives. Incorporating these ratings into electronic health records with explicit outputs that delineate the primary criteria influencing the suggestion enhances professional confidence and patient comprehension.

For successful future clinical translation, regulatory harmonisation and uniform reference ranges are essential. Implementation studies must include cost-effectiveness, compatibility with electronic health records, and the reproducibility of analytical findings. EV tests have to be included into conventional ART procedures, which include uterine fluid analysis before transfer for receptivity verification, follicular sample during retrieval for oocyte competency assessment, and blood or menstrual collection on cycle day 2 for baseline evaluation. This stratified EV-based approach has the potential to revolutionise the IVF procedure by substituting arbitrary timing assessments with molecular precision milestones. This would enhance success rates while reducing the number of cycles and psychological strain. Health-economic models demonstrating that minor enhancements in the per-cycle live birth rate and a reduction in needless transfers provide cost savings and expedite the time to pregnancy strengthen the argument for reimbursement and broader use. Crucial actions on the translational roadmap include conducting prospective, multicentre validation studies with definitive objectives (live birth, sustained implantation) and collaborating with diagnostic businesses to develop kit-based, CLIA-compliant tests.

## 8. Discussion

This narrative review constitutes the inaugural comprehensive synthesis of the burgeoning research on EVs in endometriosis-associated infertility, integrating molecular mechanisms, biofluid-specific signatures, and reproductive outcomes within a unified translational framework. Our synthesis reveals that EV cargo actively modifies the peritoneal, endometrial, and ovarian microenvironments, while simultaneously reflecting the molecular profile of endometriotic diseases. All examined biofluids, including serum, plasma, menstrual blood, follicular fluid, uterine fluid, and peritoneal exudates, revealed that EVs encompass miRNAs, proteins, and lipids that intersect with essential pathogenic signalling pathways such as PI3K/AKT/mTOR, TGF-β/SMAD, MAPK/ERK, NOTCH, and NF-κB/JAK/STAT. These molecular networks elucidate the processes through which systemic reproductive failure arises from localised pelvic inflammation via the control of fibroblast activation, macrophage polarisation, oxidative metabolism, and progesterone resistance. From a diagnostic and prognostic standpoint, certain EV-associated miRNAs, such as miR-22-3p, miR-320a, miR-145-5p, miR-21-5p, miR-23a, miR-29c, and miR-4443, proficiently differentiated between illness occurrence and ART results. Multiple investigations demonstrated AUC values beyond 0.80. EVs from serum and plasma revealed systemic disease activity. EVs from menstrual blood exhibited eutopic endometrial remodelling; uterine fluid EVs suggested implantation readiness. Follicular fluid EVs represented altered granulosa-oocyte communication. The molecular patterns demonstrated a substantial association with clinical outcomes, such as blastocyst quality, implantation rate, live birth, and metaphase-II oocyte yield, suggesting that EVs may function as comprehensive, non-invasive indicators throughout the reproductive continuum.

### 8.1. Integrative Overview and Mechanistic Convergence

A consensus is emerging that endometriosis is a vesicle-mediated multisystem illness characterised by hormone resistance, metabolic reprogramming, and chronic inflammation that extends beyond the ectopic less [199]. Multiple studies have recognised EVs as the primary biological conduits linking these activities. They serve as messengers, amplifiers, and indicators of molecular disease inside the pelvic cavity and throughout the reproductive axis. EVs transport several substances, including miRNAs, lncRNAs, proteins, lipids, and metabolites. These chemicals work together to regulate angiogenesis, oxidative stress, immunological balance, and cell cycle control [125].

Khalaj et al. (2019) characterised EVs from plasma and peritoneal fluid of women with endometriosis, revealing a disease-specific cargo enriched with inflammatory cytokines, glycolytic enzymes, and angiogenic proteins, representing the first evidence of this cross-compartmental signalling [139]. Their functional experiments demonstrated that these EVs were internalised by macrophages and endometrial stromal cells, resulting in cell adhesion, migration, and proliferation, and activated TGF-β/SMAD, STAT3, and NF-κB signalling pathways. This discovery indicated that EVs operate as active molecular relays instead of just passive byproducts. Nazri et al. (2023) published similar findings, indicating that lesion-derived EVs facilitate tolerance to hypoxia and increase glycolytic reliance by conveying metabolic and immunological signals through miRNAs that target the PI3K/AKT and HIF-1α pathways [74].

Subsequent clinical investigations directly associated these vesicular pathways with reproductive outcomes. Certain serum-EV miRNAs (miR-22-3p, miR-320a, let-7b-3p) may forecast pregnancy and delivery in IVF cycles with an AUC over 0.8 [200]. Their expression exhibits transcriptional patterns derived from lesions, as shown by Muraoka et al. (2024) [51,200]. These miRNAs together regulate the genes NOTCH1, IGF1R, and VEGF-A, essential for folliculogenesis and endometrial receptivity. Li et al. (2024) expanded these results to implantation biology, demonstrating that UF-EVs enriched in miR-145-5p inhibit NOTCH-dependent trophectoderm differentiation, thereby hindering blastocyst attachment and development [31]. This paradigm offers clear molecular continuity between the biology of endometriotic lesions, the composition of circulating EVs, and embryonic competence, in contrast to prior models that analysed these compartments in isolation.

Oxidative stress, fibrosis, and inflammation mediated by extracellular vesicles have been comprehensively described in many biofluids. Ji et al. (2024) identified miR-4443 as a crucial regulator of ACSS2/PI3K-AKT-mTOR signalling in MB-EVs [174]. The metabolic reconfiguration connects histone acetylation and acetate metabolism, hence promoting matrix deposition and fibroblast activation. Aligned with impaired differentiation and redox imbalance, Gurung et al. (2025) and Vaiciuleviciute et al. (2025) performed complementary proteomic analyses that revealed more than 5000 differentially expressed MB-EV proteins, encompassing reduced cytoskeletal regulators (ACTN1, VIM) and oxidative-stress enzymes (PRDX2, SOD1) [35,122]. The expression levels of COL1A1, FN1, and ACTA2 were elevated in naïve endometrial stromal cells subjected to these EVs, reflecting the fibrotic remodelling seen in ectopic lesions [201].

There is more evidence of EV-mediated immunological remodelling in the peritoneal environment. Research conducted by Wagner et al. (2024) demonstrates that vesicles derived from bacteria present in peritoneal exudates trigger TLR2/4-NF-κB signalling, resulting in elevated levels of IL-6 and TNF-α, hence exacerbating nociceptive hypersensitivity [202]. MiR-21-5p, miR-146a, and miR-155 established immunoregulatory microRNAs that promote M2 polarisation in macrophages and suppress cytotoxic activity of NK cells are abundant in concurrent human extracellular vesicle research. All these factors collaborate to maintain a perpetually inflammatory milieu, hindering immune system surveillance and promoting angiogenesis and neurogenesis [203].

The vesicular paradigm is similarly persuasive in the ovarian compartment. Duval et al. (2024) indicate that FF-EVs in endometriosis exhibit varying amounts of miRNAs that regulate communication between cumulus cells and oocytes, as well as mitochondrial biogenesis (miR-23a, miR-29c, and miR-125b) [37]. These alterations resemble the oxidative-stress phenotype described by Han et al. (2024) [198]. This trait has aberrant EV cargo that reduces metaphase-II yield and diminishes granulosa cell viability. Systemic inflammatory EVs emitted by lesions or peritoneal macrophages may account for these ovarian EV modifications, so connecting localised disease to overarching reproductive failure [37].

Apostolov et al. (2025) revealed that UF-EV transcriptomes at the endometrial interface reflect the cyclical gene-expression pattern of the endometrium, especially during the mid-secretory phase [32]. During non-receptive phases, miR-145-5p and miR-143-3p exhibited upregulation, while receptive phases were characterised by an abundance of EVs including miR-30d, miR-200b, and miR-375 [204]. These alterations corresponded to the amounts of adhesion and immune-modulatory molecules (ITGB3, LIF, IL1B) and were directly associated with the efficacy of implantation. The notion of a multi-compartment vesicular continuum, in which EV cargo regulates the preparedness of ovarian, embryonic, and endometrial tissues, is supported by the synthesis of these uterine findings with serum and MB-EV profiles [205].

Systems techniques have started to clarify the interconnections of EV-mediated pathways across the reproductive axis, beyond individual fluid research. Cao et al. (2024) assert that the PI3K/AKT/ERK-regulated miRNA clusters identified in serum EVs are also present in medulloblastoma extracellular vesicles (MB-EVs) [206]. This indicates that there is reciprocal transport between the endometrial and systemic circulation. Gu et al. (2003) emphasised the importance of vesicles in oxidative stress and angiogenesis via the transport of exosomal HSP70, VEGF, and Annexin A2 [96]. Zhang et al. (2024) showed that these similar miRNA protein signatures might affect ferroptotic sensitivity and mitochondrial respiration, therefore connecting vesicular signalling to the overarching redox environment that determines oocyte quality.

All of these investigations show a notable mechanistic convergence. EV cargo consistently targets molecular pathways that govern inflammation, metabolism, oxidative stress, fibrosis, and angiogenesis pathways that also regulate follicular competence, embryo development, and endometrial receptivity irrespective of biofluid or illness state [25]. Endometriosis functions as an ecosystem propelled by vesicles, whereby target tissues serve as “translational receivers” while lesion-derived extracellular vesicles operate as “molecular broadcasters.” Extracellular vesicles clarify the process by which a localised disease causes systemic infertile characteristics via the transfer of proteins and microRNAs that simultaneously maintain lesion survival and modify distant reproductive microenvironments [207].

This integrated concept, underpinned by cross-biofluid consistency and multi-omic datasets, positions extracellular vesicles as both guardians and influencers of reproductive health. Their combined function as clinically recognisable indicators and pathogenic mediators offers a unique potential for mechanism-based diagnostics and treatments. The integration of molecular pathology and clinical correlation across much independent research endorses a paradigm change. Endometriosis-related infertility is increasingly acknowledged as a systemic, vesicle-coordinated communication issue that may be interpreted, monitored, and eventually treated, rather than just a result of anatomical distortion [32,35,37,51,139,174].

### 8.2. Translational Implications for IVF and Biomarker Development

The integration of EV biology into reproductive medicine has revolutionised doctors’ methodologies for diagnosing, categorising, and managing infertility in women with endometriosis [158]. Conventional indicators like CA-125, AMH, or ultrasonographic staging do not accurately represent the dynamic, molecular-level disturbances that govern follicular competency and endometrial receptivity [98]. EVs, conversely, possess real-time biological signals from the endometrium, ovary, and immune-peritoneal interface. These tissues are important for implantation. Their molecular payload indicates the presence of the illness and its mechanisms, which may be used to forecast therapy efficacy, monitor its effectiveness, and customise ART regimens.

Recent study has shown definitive proof-of-concept. Muraoka et al. (2024) established that serum-derived small EVs containing miR-22-3p, miR-320a, and let-7b-3p may forecast pregnancy and delivery outcomes in IVF patients with endometriosis, attaining AUC values above 0.80 [51]. These miRNAs link circulating EV patterns to reproductive function by affecting essential regulators of folliculogenesis, steroidogenesis, and implantation, such as IGF1R, VEGF-A, and NOTCH1. Li et al. (2024) discovered that UF-EVs enriched in miR-145-5p inhibited embryonic NOTCH signalling, hence altering blastocyst development both in vitro and in vivo [31]. This study demonstrates that EVs may affect receptivity states via direct molecular communication between the embryo and the endometrium, rather than passively reflecting them. Regarding translation, this indicates that UF-EV miRNA panels may serve as “molecular biopsies” that are non-invasive prior to embryo transfer, either as a substitute for or in conjunction with invasive endometrial receptivity assessments.

EVs from follicles and menstrual blood provide distinct insights on oocyte and endometrial preparedness, both critical criteria for successful IVF, in addition to endometrial receptivity. Duval et al. (2024) identified atypical FF-EV cargo in women with endometriosis, notably the downregulation of mitochondrial-regulatory microRNAs (miR-23a, miR-29c, and miR-125b), which was associated with suboptimal embryo shape and reduced metaphase-II output [37]. Ji et al. (2024) report that menstrual-blood extracellular vesicles enriched in miR-4443 activate the ACSS2/PI3K-AKT-mTOR pathway [80]. This route was associated with both the severity of symptoms and the recurrence of the illness. These results indicate that extracellular vesicles may connect cellular metabolism and hormone physiology, resulting in quantifiable IVF outcomes. While FF-EV analysis may aid in formulating oocyte retrieval techniques or antioxidant supplements, profiling MB-EVs may allow doctors to assess the cyclical endometrial milieu before controlled ovarian stimulation.

Moreover, EVs may function as longitudinal and predictive indicators of therapy response. In contrast to static histological or genetic indicators, the release of extracellular vesicles is dynamic and may be influenced by medication, oxidative stress, and inflammation [85]. The production of lesion-derived extracellular vesicles is anticipated to alter as GnRH agonist levels decrease or when the lesion is excised laparoscopically. Monitoring EV cargo normalisation might function as a real-time indication of molecular remission. This will promptly influence the timing of embryo transfer after surgery or medical intervention, a subject that now lacks evidence-based agreement [208]. From a bioengineering perspective, extracellular vesicles may serve as biomarkers, medicinal agents, or delivery vehicles. EVs originating from MSCs, such as MenSC-EVs, have pro-regenerative and immunomodulatory characteristics. Vaiciuleviciute et al. (2025) and Wang et al. (2025) assert that MenSC-EVs possess more regenerative capacity, have a reduced likelihood of eliciting an immunological response, and contain a higher concentration of anti-inflammatory miRNAs and growth factors compared to bone-marrow-derived EVs [122,138]. Employing these vesicles in personalised IVF therapies may improve endometrial receptivity or reduce oxidative stress in granulosa cells by recalibrating cytokine and angiogenesis profiles. Initial preclinical investigations have shown that modified extracellular vesicles containing anti-fibrotic microRNAs (such as miR-214-3p and miR-30c-5p) may inhibit lesion progression and restore implantation capability in murine models. This substantiates the notion that vesicle-based therapies may serve as adjuncts to ART [209].

We must consider the technological and legal challenges that EV biomarkers may encounter in therapeutic applications. Quantitative variability is an issue because of the impact of hormonal confounders, isolation techniques, and pre-analytical processes on yield and composition. Adherence to the MISEV2023 criteria (as delineated by Théry et al., ISEV) is crucial for ensuring reproducibility and comparability across research, necessitating the standardisation of pre-analytical parameters (sample time, hormonal phase, isolation platform). Integrating transcriptomic, proteomic, and metabolomic data from EVs with machine learning and multi-omics approaches may improve prediction models by differentiating genuine biological signals from noise. To establish EVs as dependable clinical-grade biomarkers, future frameworks must include cross-biofluid validation (serum, menstrual blood, UF), standardised normalisation procedures, and multi-centre validation, as proposed by Apostolov et al. (2025) and Merino-Pérez et al. (2025) [32,87].

### 8.3. Molecular Interactions Between Hormonal Signalling and Extracellular Vesicle Pathways in Assisted Reproductive Technology

Oestrogen exposure promotes extracellular vesicle biogenesis at the lesion site via ESR1/ESR2-mediated regulation of RAB and ESCRT machinery, hence promoting the release of pro-inflammatory and pro-angiogenic vesicles. Investigations by Nazri et al. (2023) reveal that oestradiol stimulation induces the selective encapsulation of estrogen-responsive miRNAs, including miR-200b and miR-21-5p, within EVs, thus promoting vascular remodelling and epithelial–mesenchymal transition [74]. Estrogen-conditioned extracellular vesicles subsequently impede natural killer cell cytotoxicity and reinforce local macrophage M2 polarisation in a paracrine fashion, creating a self-sustaining estrogen extracellular vesicle feedback loop that promotes lesion development and immunological tolerance [210]. Simultaneously, EVs containing HDACs, DNMTs, and miRNAs (miR-29c, miR-125b) that inhibit progesterone receptor target genes induce progesterone resistance. This occurs due to a decrease in PR-B expression and alterations in co-regulator recruitment. Progestogenic silencing and oestrogenic amplification work together to provide a hormonal milieu that allows fibrotic and inflammatory pathways to function without obstruction [211].

Besides lesions, these endocrine extracellular vesicle interactions affect follicular physiology and oocyte competence, especially under controlled ovarian stimulation in in vitro fertilisation. Duval et al. (2024) and Han et al. (2024) assert that FF-EVs include miRNAs and metabolites that facilitate the action of FSH and LH on granulosa cells in the vicinity [37,198]. In endometriosis, aberrant extracellular vesicle profiles are marked by the overexpression of oxidative stress-related miR-100 and miR-148a, and the downregulation of miR-23a and miR-125b. These alterations interfere with the PI3K–AKT–mTOR and AMPK pathways, leading to premature luteinisation, modified mitochondrial metabolism, and decreased MII oocyte production [212]. These miRNAs are significant since they alter the equilibrium of steroidogenic enzymes and aromatase expression, hence recalibrating the production of oestradiol inside the follicle. FF-EVs release miRNAs into the bloodstream, establishing a systemic endocrine signature that may alter the priming of the endometrium and the communication between the hypothalamus and pituitary gland. This establishes an “extended hormonal axis” composed of hormone-sensitive extracellular vesicle cargo and hormones [213].

Molecular reprogramming in the endometrium, facilitated by extracellular vesicles, modulates cell responses to steroid and gonadotropin signals during the implantation window. Apostolov et al. (2025) revealed that UF-EVs, which convey messenger and non-coding RNAs that respond to variations in progesterone and oestradiol, mirror the transcriptome changes in the endometrium over the menstrual cycle [32]. In the mid-secretory phase, UF-EVs have elevated levels of miR-30d-5p and miR-200b-3p, which activate immunomodulators (LIF, IL-15) and adhesion molecules (ITGB3, MUC1), therefore establishing a vesicular signature indicative of receptivity [214]. In contrast, patients with endometriosis have dysregulated UF-EV profiles marked by reduced miR-200b-3p and increased miR-145-5p levels, resulting in the inhibition of NOTCH1 and HOXA10, two genes essential for trophoblast invasion and decidualisation. In the endometrial milieu, these extracellular vesicle patterns encode a “molecular progesterone resistance”: vesicular disruption of downstream signalling impedes intracellular receptivity programmes, despite adequate blood progesterone levels [31].

The responsiveness of vesicles and polymorphisms in the FSH receptor represents another significant hormonal interaction. Variants associated with altered follicular dynamics, such as FSHR Ser680Asn, may change the profile of EV secretion. Initial data from translational research suggest that women with the Ser/Ser genotype have increased amounts of EV-miRNAs modulated by FSH and CART, which are linked to energy metabolism and granulosa cell proliferation. The variability in ART response across people, where identical stimulation methods yield varied oocyte results, may be partly explained by this genotype-dependent EV release. Thus, investigating the genotype EV hormone axis may provide mechanistic insights into customised stimulation techniques [215].

Ultimately, EVs demonstrate hormonal action. Numerous investigations, such as those by Vaiciuleviciute et al. (2025) and Wang et al. (2025), demonstrate that extracellular vesicles produced from MenSC-EVs include growth factors and lipid mediators that may mimic local endocrine signalling [122,158]. MenSC-EVs include bioactive constituents, including prostaglandins, sphingolipids, and steroid metabolites, which function analogously to hormones, facilitating stromal differentiation, angiogenesis, and immune recruitment. The therapeutic potential of modified EVs as paracrine hormone analogues has been proven by experimental restoration of receptivity in models of endometrial damage and Asherman’s disease, using their regenerative properties.

### 8.4. Therapeutic Horizons: Targeting or Harnessing Extracellular Vesicles in Endometriosis and IVF

Therapeutic efforts have moved from symptomatic hormonal control to molecular interception and vesicular re-engineering as the mechanistic significance of EVs in endometriosis and infertility becomes undeniable. In order to restore reproductive homeostasis, the translational horizon now includes two interconnected strategies: (1) recalibrating or blocking pathogenic vesicle signalling, and (2) using vesicles as biologic drugs or delivery vectors. According to preclinical research, EV release can be controlled to slow the disease’s molecular spread. Through ceramide-dependent machinery, Nazri et al. (2023) showed that endometriotic stromal cells generate an excessive number of small EVs in response to oestrogenic stimulation [74]. In vitro, agents like manumycin A (a Ras-farnesylation inhibitor) and GW4869 (a neutral-sphingomyelinase inhibitor) significantly decreased EV secretion and downstream angiogenic signalling. Vesicular blockade decreased lesion vascularisation and IL-6/TNF-α output, according to Wagner et al. (2024)’s complementary animal data [202]. These results highlight a cross-disciplinary translational pipeline and are consistent with oncology-derived insights that exosome inhibitors reduce metastatic spread. Crucially, Cao et al. (2023) found that resveratrol and curcumin alter the activity of nSMase2 and Rab27a/b, indicating that dietary polyphenols may function as low-toxicity vesicular modulators. Even though they are early, these treatments present the idea of vesicular pharmacotherapy, which is the intentional suppression of harmful intercellular communication without changing the levels of hormones in the body [216].

The use of therapeutic EVs to restore immunological and hormonal balance is the second and possibly more revolutionary approach. Menstrual blood-derived mesenchymal stem-cell EVs (MenSC-EVs) have been described by Vaiciuleviciute et al. (2025) and Wang et al. (2025) as powerful anti-inflammatory and pro-regenerative agents [122,158]. These vesicles contain miR-146a, miR-223, and miR-21-3p, which enhance VEGF-C-driven micro-angiogenesis, reduce fibrotic gene expression, and inhibit NF-κB and STAT3 activation in macrophages. MenSC-EV treatment normalised oxidative-stress indices (SOD1, GPX4), restored PR-B expression, and decreased lesion area by more than 60% in mice with endometriosis. Proteomically defined MenSC-EVs also reduce α-SMA deposition in fibrotic endometrium and restore cytoskeletal integrity (ACTN1, VIM), according to Gurung et al. (2025) [35]. This paradigm was further extended by Sadati et al. (2024) to exosomes from MSCs derived from adipose and bone marrow, which improve granulosa cell mitochondrial function and reduce lipid peroxidation in oxidative follicular models [72]. By re-establishing oocyte competence and receptivity through molecular rather than endocrine recalibration, these studies collectively define a therapeutic class of regenerative vesicles that can function as paracrine surrogates for steroid hormones.

Vesicle-based targeted delivery and “re-education” techniques EVs are perfect for targeted molecular delivery because of their inherent biocompatibility and capacity to pass through biological barriers. Selective accumulation in lesion stroma and activated endothelium is made possible by functionalisation with integrin-binding peptides (RGD motifs), anti-CD44 antibodies, or folate ligands [217]. Vesicles loaded with miR-214-3p and miR-30c-5p inhibited TGF-β1, COL1A1, and CCN2, reversing matrix accumulation in fibrotic models. Similar designs are being tested to deliver siRNAs targeting HIF-1α or VEGFA, which are pathways frequently highlighted in Nazri et al. (2023) and Gu et al. (2003), or antioxidants (N-acetylcysteine, melatonin) [74,96]. These engineered exosomes can now be produced in large quantities under GMP conditions thanks to parallel developments in lipid-fusion and microfluidic electroporation techniques. “EV re-education,” or pharmacologically guiding the content of endogenous EVs towards advantageous signatures, is a concept that is gaining popularity. While high progesterone redirected endometrial EVs from miR-145-5p towards miR-200b, restoring the NOTCH–LIF implantation axis described by Li et al. (2024), Han et al. (2024) showed that FSH stimulation changed granulosa-cell EV cargo to enhance mitochondrial gene expression (PGC1A, TFAM) [31,198]. These results present a feedback-sensitive framework that allows for the optimisation or monitoring of therapeutic endocrine modulation through changes in EV profiles.

Systemic interventions may indirectly recalibrate EV signalling because vesicle output reflects oxidative and metabolic stress. Serum-EV miR-22-3p and miR-320a levels were linked to IVF success by Muraoka et al. (2025), indicating that pre-treatment with antioxidant or anti-inflammatory drugs may normalise these biomarkers [51]. Ferroptosis inhibitors and iron chelators decrease the release of lipid-peroxidation-enriched EVs in granulosa-cell cultures, according to evidence from Zhang et al. (2024) [218]. Melatonin supplementation, on the other hand, improved MII yield by modulating FF-EV cargo towards mitochondrial and anti-apoptotic miRNAs, according to Duval et al. (2024) [37]. These findings collectively imply that pharmacologic and dietary interventions can act as non-invasive vesicular regulators, bridging the gap between molecular precision therapy and lifestyle change.

The creation of multiomic and AI-assisted pipelines that link EV molecular signatures to treatment outcomes is a crucial facilitator of these advancements. Gurung et al. (2025) used network-based correlation to predict oxidative-stress responses from MB-EV proteomes, while Cao et al. (2023) and Apostolov et al. (2025) used integrative analyses using machine learning classifiers to distinguish receptive from non-receptive UF-EV profiles with >90% accuracy [32,35,216]. The EV-guided ART protocols, which use vesicle metrics to guide cycle initiation, gonadotropin dosage, and embryo-transfer timing, are made possible by these computational frameworks. Strict adherence to ISEV Reproductive EV Task Force and MISEV2023 standards which address isolation, quantification, and nomenclature is necessary to guarantee translational fidelity [219]. Therapeutic EVs are already categorised as biologic medicinal products by regulatory bodies, which require potency tests, stability information, and a genomic-safety assessment before human use. Table 3 delineates the most sophisticated treatment approaches using EVs as pharmacological targets or biological delivery systems in endometriosis and ART. It illustrates how mechanistic targeting may lead to regenerative applications.

A concise overview of the translational and therapeutic uses of EVs in reproductive medicine and endometriosis. Research on EVs as therapeutic agents or instruments for biomarker-guided interventions includes both experimental and first translational models. MSC denotes mesenchymal stem cell, MenSC signifies menstrual-blood stem cell, ROS represents reactive oxygen species, mtDNA refers to mitochondrial DNA, MII indicates metaphase II oocyte, and ART stands for assisted reproductive technology.

Table 3 illustrates the progression of treatment concepts for ART and endometriosis centred on EVs from observational biology to functional regulation. A method through which bone marrow-derived MSC-EVs and MenSC-EVs exhibit consistent anti-inflammatory and antioxidative effects is via the miR-146a-mediated inhibition of TLR4 and NF-κB (Vaiciuleviciute et al., 2025; Wang et al., 2025) [122,158]. Menstrual EV-derived miR-4443 modulates acetate metabolism via ACSS2, diminishing stromal fibrosis an optimistic antifibrotic strategy, as shown by Ji et al. (2024) [174]. Under oxidative circumstances, MSC-EVs on the ovarian side restored oocyte maturation and mitochondrial activity (Duval et al., 2024), indicating that vesicle-based adjuncts may preserve follicular competence during stimulation [37]. These results indicate a significant transition towards electric vehicle-guided personalised reproductive therapies, in which vesicles function as therapeutic messengers and diagnostic sensors. The inherent targeting capability, biocompatibility, and ability to transport numerous functional molecules of EV treatments enhance their translational feasibility. Prior to clinical use, extensive testing, standardisation of GMP, and immunological safety profiling remain essential.

The absence of defined protocols for the collection, characterisation, and quantification of EVs is a significant obstacle impeding the clinical progression of EV-based biomarkers in reproductive medicine. Current investigations use several procedures, including differential ultracentrifugation, size-exclusion chromatography, precipitation-based kits, and microfluidic platforms. These factors may result in varying assessments of EV purity, concentration, and biomarker repeatability. Comparisons among studies and clinical interpretations are further hampered by discrepancies in cycle-phase timing, sample processing, pre-analytical storage, and normalisation procedures.

To bridge this gap, it is essential to implement standardised operating procedures that adhere to MISEV requirements and rigorous quality controls both internally and externally. It is essential to establish standardised procedures for the processing of follicular fluid and uterine fluid EVs, conduct multi-centre research using identical isolation and sequencing protocols, and confirm EV-miRNA signatures across laboratories. To transition from exploratory findings to clinically applicable EV-based diagnoses in ART environments, fundamental criteria must be established.

Despite the increasing evidence that EV-based markers may differentiate between endometriosis and healthy reproductive states, direct comparisons with other inflammatory or gynaecological disorders are absent. Claims about diagnostic specificity should be regarded as tentative until meticulously structured comparison studies are conducted, notwithstanding the encouraging present results.

Hormonal stimulation during IVF alters the dynamics of follicular and systemic signalling, including the composition and secretion rates of extracellular vesicle cargo. Ovarian stimulation is a significant biological and methodological confounder that must be accounted for in study design and biomarker interpretation frameworks.

Robust clinical and scientific evidence supports the notion of uterine-fluid extracellular vesicle profiling just before embryo transfer. A variety of technological and procedural challenges must be resolved prior to practical implementation. These include atraumatic sampling, cycle-timing precision, laboratory standardisation, rapid analytical turnaround, and patient acceptance. These concerns highlight the need of stringent staged development before inclusion into clinical practice.

## 9. Challenges, Constraints, and Prospects

The use of EV research in the therapeutic treatment of endometriosis-related infertility is still in its early, experimental stages, despite the significant advancements outlined in this paper. Prior to the incorporation of EV-based tests or therapies into ART processes, significant technological, biological, and regulatory obstacles must be addressed, despite the rapid growth of the molecular and diagnostic evidence base. Future advancement necessitates standardisation, repeatability, and collaboration across disciplines such as reproductive endocrinology, molecular biology, and bioengineering.

A significant issue is the absence of standardised protocols for isolating, quantifying, and characterising EVs. The centrifugation velocities, filtration thresholds, and precipitation techniques used in current research demonstrate considerable variety, resulting in inconsistencies in particle yield, size distribution, and cargo purity [220]. Nazri et al. (2023) employed density-gradient and differential ultracentrifugation techniques for isolating lesion and peritoneal EVs, while Muraoka et al. (2024) and Apostolov et al. (2025) utilised size-exclusion chromatography and NTA for serum and uterine-fluid EVs [32,51,74]. These methodological discrepancies complicate result reproduction and hinder meta-analyses. The interpretation of data is further confounded by variations in sample scheduling about the menstrual cycle, hormone medication, or ovarian stimulation. To guarantee the validity of cross-study comparisons, pre-analytical factors, including luteal vs. follicular phase sample, ART cycle day, and fasting state, must be standardised. Multi-centre biobanking initiatives, such as those suggested by the ISEV Reproductive Task Force and Vaiciuleviciute et al. (2025), need to establish reference biofluids, standard operating procedures, and calibration benchmarks for extracellular vesicle-based reproductive biomarkers [122].

Endometriosis is a complex illness affected by environmental, genetic, and epigenetic variables, all of which may alter EV markers. Causal inference is hindered by the restricted sample sizes and cross-sectional designs of several studies, including Ji et al. (2024) and Khalaj et al. (2019) [139,174]. The impact of exogenous hormones (oral contraceptives, GnRH analogues, progesterone support) and comorbidities (obesity, thyroid dysfunction, metabolic syndrome) on vesicular cargo composition remains ambiguous. Analogous to oxidative stress and BMI, these factors can modify the kinetics of EV release and the miRNA composition. Redox imbalance influences the miR-29 and miR-100 families, producing interference that may obscure disease-specific markers, as reported by Sadati et al. (2024) [72]. Longitudinal, paired-sample designs that record EV dynamics before and after hormone treatment, surgery, or stimulation will be crucial for distinguishing signal from background fluctuation. The predicted accuracy might be significantly improved by creating covariate-adjusted bioinformatic models that include age, BMI, cycle phase, and genotype (e.g., FSHR Ser680Asn, eNOS rs2070744).

Numerous studies have shown that EV miRNA panels, including miR-22-3p, miR-320a, and miR-145-5p, exhibit good diagnostic accuracy (Muraoka 2025; Li 2021) yet, none have been subjected to prospective, blinded, multicentre validation. For clinical adoption, tests must be repeatable, capable of accommodating pre-test variations, and provide additional value to existing assessments such as AMH, CA-125, progesterone assays, and ultrasound [51,142]. Regulatory approval necessitates the establishment of limitations on biological and analytical variability, similar to those required for endocrine biomarkers. To prevent false positives resulting from variations in plasma volume during ovarian stimulation, Duval et al. (2024) and Gurung et al. (2025) emphasise that extracellular vesicle quantification must be normalised not only by particle count but also by protein or RNA mass [35,37]. The cost, turnaround time, and need for ultracentrifugation are complicating access. Recent immunoaffinity-capture and microfluidics technologies, shown by Cao et al. (2023), have the potential to democratise extracellular vesicle diagnoses by enabling same-day analyses that align with IVF clinic timelines [216].

Therapies based on extracellular vesicles continue to face significant challenges regarding target selectivity, scalability, and biosafety. MenSC-EVs and MSC-EVs have shown their capacity to combat inflammation and enhance cellular receptivity (Vaiciuleviciute 2025; Wang 2025), although ensuring their purity and stability during large-scale production remains challenging [122,158]. The diversity of EV cargo raises concerns for off-target consequences, including accidental modifications of angiogenic or carcinogenic pathways. The European Medicines Agency currently categorises exosome treatments as biological medical goods. This necessitates adherence to GMP, potency assessments, and genetic stability evaluations. Furthermore, transgenerational safety requires thorough assessment, especially in ART populations, since EVs may transfer microRNAs and epigenetic regulators to germ cells. Longitudinal offspring data are inadequate. Nevertheless, preclinical reproductive toxicology investigations conducted by Han et al. (2024) have not shown any evidence of teratogenicity or chromosomal damage [198]. Ethical issues include informed permission for embryonic stem cell harvesting, particularly from follicular aspirates or menstrual blood, as well as the fair allocation of innovative therapeutics.

In the subsequent phase, descriptive research needs to be replaced by integrative and interventional research. By employing multiomics methodologies that integrate vesicular miRNA, proteomic, lipidomic, and metabolomic profiles, it may be feasible to delineate the whole “EV connectome” associated with infertility related to endometriosis. This integration would elucidate the convergence of endocrine, metabolic, and systemic inflammatory inputs on vesicular signalling networks. Research combining single-cell transcriptomics with extracellular vesicle tracking may clarify the bidirectional interactions among granulosa cells, stromal fibroblasts, and immune populations, as shown by Apostolov et al. (2025) and Duval et al. (2024) [32,37]. The integration of EV analysis into ART trial design in clinical settings might transform the evaluation of embryo-transfer readiness, cycle scheduling, and patient selection. For instance, normalising serum extracellular vesicle microRNA post-surgery may assist in determining the optimal initiation of in vitro fertilisation, while real-time ultrafiltration extracellular vesicle monitoring may aid in identifying the most advantageous timing for implantation. Ultimately, ART may be tailored to each woman’s unique molecular profile by EV-based companion diagnostics, which are genetic tests connected to cancer.

## Figures and Tables

**Table 1:** Extracellular vesicle–derived biomarkers associated with ART outcomes in women with endometriosis.

First Author (Year)	EV Source (Matrix)	Key EV Cargo (miRNA/Protein)	Major Pathway/Target	Functional Outcome	ART Parameter Correlated	Notes/Validation
Muraoka et al., 2024, [51]	Serum EVs,	miR-22-3p, miR-320a	IGF1R, VEGFA, angiogenic regulation	Promotes implantation competence	↑ Clinical pregnancy, ↑ live birth (AUC > 0.8)	ROC-based discrimination between pregnant vs. non-pregnant
Li et al., 2024, [31]	Uterine-fluid EVs,	miR-145-5p	NOTCH1 pathway repression	Impaired blastocyst formation	↓ Blastocyst rate, ↓ implantation	Direct inhibition of embryo development confirmed in vitro
Duval et al., 2024, [37]	Follicular-fluid EVs,	miR-23a, miR-29c, miR-125b	PI3K/AKT and mitochondrial biogenesis	Reduced granulosa cell support and oxidative balance	↓ MII oocytes, ↓ embryo quality	Reflects altered follicular microenvironment
Apostolov et al., 2025, [32]	Uterine-fluid EVs,	miR-30d, miR-200b, miR-375	LIF and integrin-mediated adhesion	Optimises window of implantation	↑ Implantation and CPR	Multi-omics validation with endometrial tissue
Sadati et al., 2024, [72]	Plasma EVs,	miR-451a, miR-148a, miR-23b	Mitochondrial regulation, energy metabolism	Enhanced oocyte competence	↑ Fertilisation, ↑ embryo morphology scores	Supported by integrative bioinformatics
Brady et al., 2024, [73]	Plasma EVs (young cohort)	miR-542-3p, let-7b-3p, miR-548i	Cell cycle and hormonal modulation	Predictive for early endometriosis	AUC = 0.77 for diagnostic model	Hormone use-adjusted neural network model
Nazri et al., 2023, [74]	Peritoneal EVs	VEGF-A, MMP-2	Angiogenesis, ECM remodelling	Lesion vascularization	Correlates with pain and poor implantation	Mechanistic confirmation in macrophage cultures
Gurung et al., 2025, [35]	Menstrual-blood EVs	α-SMA, COL1A1, FN1	TGF-β/SMAD2/3, fibrosis	Fibrotic remodelling impairs receptivity	↓ CPR, ↑ dysmenorrhea	Proteomic dataset > 5000 proteins
Wang et al., 2025, [75]	MenSC-EVs (experimental)	miR-146a, miR-223	NF-κB inhibition, anti-inflammatory	Improved endometrial receptivity	Restored implantation in murine model	Translational regenerative study

**Table 2:** Diagnostic and prognostic performance of multi-miRNA panels and biomarkers derived from extracellular vesicles in endometriosis and ART outcomes.

First Author (Year)	Matrix/EV Source	EV Biomarkers Included	Endpoint/Outcome	Model Type	AUC (95% CI)	Sensitivity (%)	Specificity (%)	Validation Cohort/Method
Muraoka et al., 2025, [51]	Serum EVs	miR-22-3p + miR-320a	Clinical pregnancy & live birth prediction	ROC logistic model	0.84 (0.77–0.92)	83	79	Internal validation, n = 48 ART pts
Brady et al., 2024, [73]	Plasma EVs	miR-542-3p, let-7b-3p, miR-548i, miR-769-5p, miR-30c-1-3p	Endometriosis diagnosis in adolescents/young adults	Neural-network classifier	0.77 (0.67–0.87)	83	58	Training/test split (2:1)
Li et al., 2024, [31]	Uterine-fluid EVs	miR-145-5p	Blastocyst developmental competence	ROC analysis	0.82 (–)	80	72	Mouse & human embryo validation
Apostolov et al., 2025, [32]	Uterine-fluid EVs	miR-30d, miR-200b, miR-375	Implantative vs. non-implantative endometrium	Logistic regression	0.91 (0.85–0.97)	86	83	Paired tissue + UF cohort
Sadati et al., 2025, [72]	Plasma EVs	miR-451a, miR-148a, miR-23b	Endometriosis diagnosis	Logistic ROC model	0.81 (0.74–0.89)	79	76	Bioinformatics + qPCR validation
Nazri et al., 2023, [74]	Peritoneal EVs	VEGF-A + MMP-2 protein ratio	Disease severity classification	Linear-discriminant model	0.80 (–)	75	78	Cross-validation (n = 70)
Gurung et al., 2025, [35]	Menstrual-blood EVs	Proteomic signature (22 proteins)	Early-stage vs. advanced endometriosis	Random-forest model	0.83 (0.76–0.90)	82	80	Proteomic dataset > 5000 proteins
Ji et al., 2024, [174]	Menstrual-blood EVs	miR-4443 ± dysmenorrhea score	Symptom severity/recurrence risk	ROC composite model	0.93 (0.88–0.98)	87	91	Independent clinical cohort

**Table 3:** Therapeutic and translational applications of EVs in endometriosis and reproductive medicine.

Study/Year	EV Source/Type	Targeted Pathway	Mechanistic Effect	Experimental Model	Observed Outcome	Translational Potential
Wang et al., 2025, [158]	Menstrual-blood stem cell–derived EVs (MenSC-EVs)	NF-κB/IL-6 axis	Downregulation of pro-inflammatory cytokines; immune modulation	Murine endometriosis model	↓ Lesion size, ↓ macrophage infiltration, ↑ receptivity markers	Cell-free regenerative therapy for endometrial repair
Vaiciuleviciute et al., 2025, [122]	MenSC-EVs	miR-146a, miR-223	Inhibition of TLR4 and TNF signalling; anti-oxidative stress	In vitro & ex vivo models	Reduced ROS, restored mitochondrial balance	Autologous menstrual stem cell EV infusion
Ji et al., 2024, [174]	Menstrual-blood EVs	PI3K/AKT/ACSS2	miR-4443-driven acetate metabolism inhibition	Human stromal cells	↓ Fibrosis, ↓ α-SMA, ↓ COL1A1 expression	Non-invasive antifibrotic marker target validation
Nazri et al., 2023, [74]	Peritoneal EVs	VEGF/HIF-1α	Anti-angiogenic modulation	Macrophage co-culture	↓ VEGFA secretion, ↓ endothelial migration	EV-targeted anti-angiogenic drugs
Duval et al., 2024, [37]	Follicular-fluid EVs	Mitochondrial/AMPK regulation	Restoration of redox homeostasis	Human granulosa cells	↑ ATP, ↑ mtDNA copy number	Biomarker-guided antioxidant co-therapy in IVF
Cao et al., 2023, [216]	Serum EVs	MAPK/ERK/Ferroptosis	Inhibition of lipid peroxidation	In vitro human endometrium	↓ Lipid ROS, ↑ GPX4	EV signature guided metabolic interventions
Muraoka et al., 2025, [51]	Serum EVs	miR-22-3p + miR-320a	Angiogenic and implantation pathway modulation	Human ART cohort	↑ Pregnancy rate	Predictive tool for individualised IVF cycles

## Data Availability

No new data were created or analyzed in this study. Data sharing is not applicable to this article.

## Abbreviations

ART	Assisted Reproductive Technology
EV	Extracellular Vesicle
miRNA	MicroRNA
FF:	Follicular Fluid
PF	Peritoneal Fluid
EMT	Epithelial–Mesenchymal Transition
MVB	Multivesicular Body
ESCRT	Endosomal Sorting Complex Required for Transport
GC	Granulosa Cell
hESC	Human Endometrial Stromal Cell
IVF	In Vitro Fertilisation
ICSI	Intracytoplasmic Sperm Injection
ER	Endometrial Receptivity
ERα	Estrogen Receptor Alpha
PR	Progesterone Receptor
IL	Interleukin
TNF-α	Tumour Necrosis Factor-Alpha
TGF-β	Transforming Growth Factor-Beta
VEGF	Vascular Endothelial Growth Factor
HIF-1α	Hypoxia-Inducible Factor-1 Alpha
